# *In situ* visualization of newly synthesized proteins in environmental microbes using amino acid tagging and click chemistry

**DOI:** 10.1111/1462-2920.12436

**Published:** 2014-04-01

**Authors:** Roland Hatzenpichler, Silvan Scheller, Patricia L Tavormina, Brett M Babin, David A Tirrell, Victoria J Orphan

**Affiliations:** 1 Divisions of Geological and Planetary Sciences, California Institute of TechnologyPasadena, CA, 91125, USA; 2 Divisions of Chemistry and Chemical Engineering, California Institute of TechnologyPasadena, CA, 91125, USA

## Abstract

Here we describe the application of a new click chemistry method for fluorescent tracking of protein synthesis in individual microorganisms within environmental samples. This technique, termed bioorthogonal non-canonical amino acid tagging (BONCAT), is based on the *in vivo* incorporation of the non-canonical amino acid *L*-azidohomoalanine (AHA), a surrogate for *l*-methionine, followed by fluorescent labelling of AHA-containing cellular proteins by azide-alkyne click chemistry. BONCAT was evaluated with a range of phylogenetically and physiologically diverse archaeal and bacterial pure cultures and enrichments, and used to visualize translationally active cells within complex environmental samples including an oral biofilm, freshwater and anoxic sediment. We also developed combined assays that couple BONCAT with ribosomal RNA (rRNA)-targeted fluorescence *in situ* hybridization (FISH), enabling a direct link between taxonomic identity and translational activity. Using a methanotrophic enrichment culture incubated under different conditions, we demonstrate the potential of BONCAT-FISH to study microbial physiology *in situ*. A direct comparison of anabolic activity using BONCAT and stable isotope labelling by nano-scale secondary ion mass spectrometry (^15^NH_3_ assimilation) for individual cells within a sediment-sourced enrichment culture showed concordance between AHA-positive cells and ^15^N enrichment. BONCAT-FISH offers a fast, inexpensive and straightforward fluorescence microscopy method for studying the *in situ* activity of environmental microbes on a single-cell level.

## Introduction

Two major overarching goals in microbial ecology are to understand the ecophysiology of microorganisms and how they react to environmental stimuli over a range of spatial and temporal scales. To achieve this, methodologies and experiments that track microbial activity in an environmental context are essential. One of the most powerful and direct approaches for deciphering structure-function relationships of microbial communities is whole-cell fluorescence microscopy. Using fluorescence *in situ* hybridization (FISH) techniques, the visualization of rRNA (phylogenetic identity), specific genes (genetic potential) and messenger RNA (mRNA; gene expression) has been demonstrated in individual microbial cells (DeLong *et al.*, 1989; Amann *et al.*, 1992; Lanoil and Giovannoni, 1997; Schönhuber *et al*., 1997; Pernthaler and Amann, 2004; Zwirglmaier *et al.*, 2004; Smolina *et al.*, 2007; Kawakami *et al.*, 2010; Moraru *et al.*, 2010; Stoecker *et al.*, 2010; Yilmaz *et al.*, 2010; Behnam *et al.*, 2012). However, due to the limitations associated with using expression of rRNA or mRNA as metabolic tracer, and the observation that, on single-cell level, mRNA and protein expression for the same gene may be uncorrelated, detection of protein synthesis is considered to be a more reliable marker of activity (Wagner *et al.*, 1995; Binder and Liu, 1998; Morgenroth *et al.*, 2000; Odaa *et al.*, 2000; Schmid *et al.*, 2001; Bollmann *et al.*, 2005; Foster *et al.*, 2009; Taniguchi *et al.*, 2010). To detect and localize target proteins involved in specific metabolisms within microbial cells, immunofluorescence analyses have been used (e.g. Lin *et al.*, 1998; Fiencke and Bock, 2004; Wrede *et al.*, 2013). However, this technique is not routinely applied in microbial ecology, likely due to the time and expense involved in antibody synthesis, and challenges in assessing and controlling for specificity in uncultured microorganisms. Additionally, immunofluorescence staining of proteins cannot differentiate the timing of protein synthesis. As such, the development of a general fluorescence microscopy method that can be used to study the expression of proteins in environmental microbes *in situ* would offer a powerful complement to the current suite of methods. *In situ* tracking of *de novo* protein synthesis in the context of the microbial community and changing ecological or physicochemical conditions would facilitate new strategies for assessing the structural and functional adaptation of microbes to environmental stimuli.

Currently, two approaches are used to monitor activity of individual cultured and uncultured microbes via the incorporation of isotopically labelled substrates. The use of stable isotopes, such as ^15^N-labelled ammonia (NH_3_) or ^13^C/^15^N-labelled amino acids can be used to identify anabolically active cells (e.g. protein synthesis) within complex samples using secondary ion mass spectrometry (SIMS) (Dekas *et al.*, 2009; Orphan *et al.*, 2009; Morono *et al.*, 2011; Pernice *et al.*, 2012) or Raman microspectroscopy (Haider *et al.*, 2010). Additionally, radioactively labelled amino acids (e.g. ^14^C-leucine or ^35^S-methionine) have been used to track uptake into cells using micro-autoradiography (MAR) (Herndl *et al.*, 2005; Sintes and Herndl, 2006; Teira *et al.*, 2006). In addition, MAR-based visualization of ^14^CO_2_ assimilation by heterotrophic bacteria has been used as a general activity marker in pure cultures and environmental samples (Roslev *et al.*, 2004; Hesselsoe *et al.*, 2005). While these approaches offer a valuable link between anabolic activity and phylogeny for environmental microbes, they also require specialized laboratory facilities (radioactive certification in the case of MAR) or expensive instrumentation (microRaman or SIMS) which limits their general application in the field.

In addition to visualizing cellular protein and RNA expression, a complementary approach for identifying active cells is based on the incorporation of the indicator dye RedoxSensor green (RSG; Invitrogen), a nontoxic fluorescent indicator of bacterial reductase activity. While the full utility of RSG for microbial ecosystems has not yet been investigated, recent publications have successfully used this assay to detect respiratory activity in cultured aerobic microbial cells and environmental samples in near real time (Kalyuzhnaya *et al.*, 2008; Konopka *et al.*, 2011; Orman and Brynildsen, 2013).

Here, we explore the use of another promising approach for investigating the function and activity of environmental microorganisms based on bioorthogonal compounds coupled with click chemistry. Bioorthogonal compounds are defined as synthetic molecules that are not biologically synthesized and that do not interfere with processes within the cell. Frequently, these compounds are analogs of native biomolecules (e.g. amino acids, nucleotides, lipids, carbohydrates) that contain a functional group that is amenable for click chemistry, most commonly using an alkyne-azide reaction, which, through cycloaddition, form a triazole conjugate (i.e. click reaction; for recent reviews, see Best, 2009; Sletten and Bertozzi, 2009; Lim and Lin, 2010).

**Fig 1:**
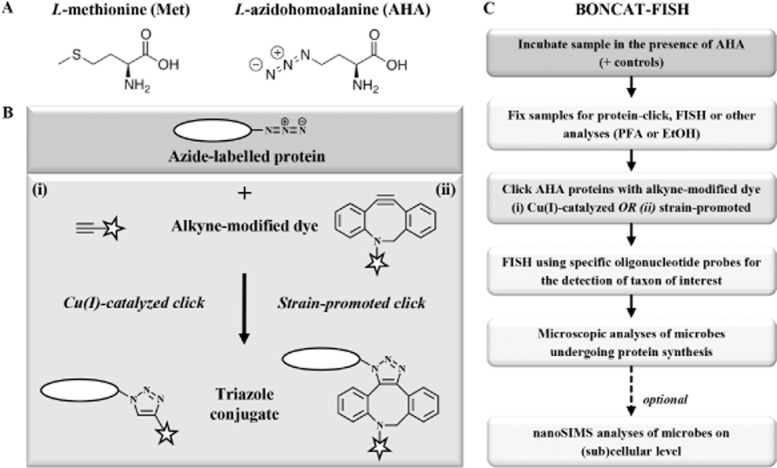
Overview of the BONCAT method for visualizing newly synthesized proteins. A. Structures of Met and its surrogate AHA, which is incorporated into newly made peptides during translation. B. The click chemistry-mediated visualization of newly produced AHA-containing proteins can be achieved via either one of two strategies: (i) in a Cu(I)-catalyzed reaction a terminal alkyne that is coupled to a fluorescence dye (star) is linked to the azide group of AHA yielding a triazole (left side); (ii) conjugation can also be achieved via strain-promoted cycloaddition, a Cu-free variation of click chemistry (right side). C. Protocol for the combinatorial labelling of AHA-containing proteins via BONCAT and rRNA-targeted FISH.

There are a series of bioorthogonal amino acids with azide or alkyne groups that have been shown to successfully compete with native amino acids and to be incorporated into the polypeptide chain during translation. However, only a subset of these non-canonical amino acids is able to exploit the substrate promiscuity of the native translational machinery without the need for genetic modification of the host cell (Ngo and Tirrell, 2011). The *l*-methionine (Met) surrogate *L*-azidohomoalanine (AHA; Fig. 1A) has been shown to be the most translationally active, with activation rates approximately 390 times lower than Met in *Escherichia coli* (compared with homopropargylglycine (HPG) and norleucine that have activation rates 500 and 1050 times lower than Met; Kiick *et al.*, 2002). The rate of AHA incorporation into new proteins is regulated by methionyl-tRNA synthetase, the enzyme that catalyzes the esterification of Met with its tRNA to form a methionyl-tRNA (Kiick *et al.*, 2002). AHA is water soluble, nontoxic and stable at physiological and most environmentally relevant conditions (with the exception of highly sulfidic, alkaline habitats; see Results section). The small difference in the molecular size of AHA compared with Met results in only minimal structural disturbance after incorporation into proteins (Kiick *et al.*, 2002; Best, 2009). AHA has been used in a range of studies targeted to decipher translational regulation in *E. coli* (Kiick *et al.*, 2002; Nessen *et al.*, 2009; Ngo *et al.*, 2009) as well as amphibian and mammalian cell and tissue cultures, including immune cells (Ngo *et al.*, 2009; Howden *et al.*, 2013), kidney cells (Dieterich *et al.*, 2006), neurons (Dieterich *et al*., 2006; 2010; Yoon *et al.*, 2012) and HeLa cells (Bagert *et al.*, 2014). None of these studies detected a detrimental effect of AHA by microscopy. Moreover, studies that combined AHA-labelling with mass spectrometric proteomic sequencing reported that incubation with up to 1 mM AHA in the growth medium did not result in the preferential synthesis or degradation of proteins in *E. coli* or different human cell lines (Dieterich *et al.*, 2006; Eichelbaum *et al.*, 2012; Bagert *et al.*, 2014). The properties of AHA suggest that the specificity of this assay for measuring new protein synthesis in environmental microorganisms is high. Furthermore, non-specific reactivity with azide moieties other than AHA is likely very low, as there is only one organism (a dinoflagellate) currently known to produce an azide-containing metabolite (Griffin, 1994). In contrast, click reactions using azide-modified probe molecules may have a greater potential for non-specific reactions with naturally occurring alkyne-containing biomolecules, such as polyynes (Shi Shun and Tykwinski, 2006).

The azide moiety of AHA makes it distinguishable from the large pool of other amino acids in a cell, allowing selective click chemistry-mediated detection of proteins that have been synthesized during AHA incubation, an approach that has been termed bioorthogonal non-canonical amino acid tagging (BONCAT; Dieterich *et al.*, 2006). The azide-alkyne reaction has a number of beneficial characteristics for use in biomolecule detection, including fast kinetics with high chemo- and regio-specificity, a single reaction product, and very high yields (Best, 2009; Lim and Lin, 2010). The assay is simple to perform under biologically relevant conditions and takes place in the presence of water or other solvents. There are two forms of azide-alkyne click reactions (Fig. 1B): (i) a Cu(I)-catalyzed cycloaddition reaction of an azide with a terminal alkyne (Huisgen, 1963; Rostovtsev *et al.*, 2002; Torne *et al.*, 2002); and (ii) a strain-promoted variant that makes use of a highly reactive strained cyclo-octyne system that allows the reaction to take place in the absence of a copper catalyst (Agard *et al.*, 2004; Codelli *et al.*, 2008). Both click reactions enable the selective conjugation of azides with alkynes within biologically and chemically complex environments (for reviews, see Carrico, 2008; Best, 2009; Sletten and Bertozzi, 2009; Jewett and Bertozzi, 2010).

Recently, an approach for the AHA-based fluorescence visualization of protein synthesis has been developed (Beatty *et al.*, 2006; Dieterich *et al.*, 2007). It allows microscopic detection of newly synthesized proteins that have incorporated bioorthogonal amino acids via click chemistry conjugated fluorescence dyes (Fig. 1B). Fluorescence visualization of BONCAT-labelled cells has been used to study protein synthesis in mammalian cell cultures and zebra fish (Beatty *et al*., 2006; 2010; Ngo *et al.*, 2009; Dieterich *et al.*, 2010; Hong *et al.*, 2010; Hinz *et al.*, 2012), as well as applied to select cultured microorganisms, i.e. *E. coli* (Beatty *et al.*, 2005; Ngo *et al.*, 2009), *Pseudomonas entomophila* (Chakrabarti *et al.*, 2012), pathogenic chlamydia (Ouellette *et al.*, 2011), and *Listeria monocytogenes* (Siegrist *et al.*, 2013). These studies reveal the general applicability of BONCAT and click chemistry in traditional model organisms; however, the suitability of this method for the study of complex, natural microbial communities *in situ* has not been explored.

Here, we outline a protocol using BONCAT for assessing metabolic activity and protein synthesis that can be combined with existing single-cell targeted approaches in microbial ecology. BONCAT was used to visualize *de novo* synthesized proteins in a range of phylogenetically and physiologically diverse bacteria and archaea as well as a series of enrichments and environmental samples. Combining BONCAT and rRNA-targeted FISH, we demonstrate the ability to directly correlate translational activity of single microbial cells with their taxonomic identity by fluorescence microscopy and study the physiology of microorganisms *in situ*. BONCAT-FISH offers an inexpensive, simple and fast approach to study anabolically active microbes via fluorescence microscopy, which has been a core method of microbial ecology for decades. It opens a new level of inquiry to researchers interested in studying the ecophysiology and *in situ* activity of uncultured microbes.

## Results and discussion

### Cu(I)-catalyzed and strain-promoted click using fluorescent and halogenated dyes

We initially performed AHA-labelling incubations with *E. coli* cultures to establish a protocol for the fluorescence labelling of AHA-containing proteins in chemically fixed cells. Labelling protocols were evaluated and updated during the study (for a non-technical overview, see Fig. 1C). AHA-containing *E. coli* cells were used to test Cu(I)-catalyzed and strain-promoted click reactions with six alkyne-modified fluorescent dyes (spanning emission wavelengths from 524 to 571 nm; Supporting Information Fig. S1). All of these dyes are commercially available alkyne derivatives of widely used fluorophores and can be analyzed using conventional fluorescence microscopy filter sets. The removal of unincorporated dyes using the washing protocol was effective for all fluorophores except Eosin (Supporting Information Fig. S1D), a bromine-containing dye that, like the fluorinated Oregon Green 488 alkyne, can theoretically be used to detect AHA incorporation into single cells via halogen ion imaging by nano-scale secondary ion mass spectrometry (nanoSIMS; see below). The inability to remove unreacted Eosin-alkyne may be due to the inherent properties of the dye, which is commonly used as a cytoplasmic stain in histological studies.

Cu(I)-catalyzed and strain-promoted click reactions were evaluated in BONCAT-labelling of cultures of *E. coli* and *Desulfovibrio alaskensis* with two chemically similar dyes, Carboxyrhodamine 110 Alkyne and DBCO-PEG_4_-Carboxyrhodamine 110 (ClickChemistryTools) at equimolar concentrations (10 μM; Supporting Information Fig. S1). While signal intensities were essentially identical, much higher signal-to-noise ratios were observed for samples analyzed with the Cu(I)-catalyzed reaction as compared with the strain-promoted process (data not shown). This difference may be due to the fact that strain-promoted click chemistry can be accompanied by nonspecific reactions with free thiols (for details on thiol-blocking, see Experimental Procedures section). Decreasing the dye concentration from 10 μM to the range typically used for strain-promoted click chemistry (0.1–1 μM) substantially increased the signal-to-noise ratio for this reaction.

Click chemistry-mediated fluorescence labelling was successful with slide-immobilized biomass as well as with samples in solution, with both producing equivalent fluorescence intensities and signal-to-noise ratios (tested with dye Carboxyrhodamine 110 Alkyne and DBCO-PEG_4_-Carboxyrhodamine 110 for both; data not shown). To increase the ease and reproducibility of sample handling during methods optimization the majority of our testing was conducted with slide-immobilized cells.

### BONCAT of physiologically and phylogenetically diverse microbes

We performed AHA incubations with several environmentally relevant pure and enrichment cultures of bacteria and archaea, representing a range of different physiologies and taxonomic affiliations, to test their ability to incorporate AHA into new proteins. Cultures included: (i) *E. coli*, as an example of a well-studied aerobic heterotrophic bacterium; (ii) *Paracoccus denitrificans*, a facultatively anaerobic denitrifying alphaproteobacterium; (iii) *Desulfovibrio alaskensis*, a deltaproteobacterial sulfate reducer (obligate anaerobe); (iv) *Methanosarcina acetivorans*, a methanogen within the *Euryarchaeota* (obligate anaerobe); (v) a propane-oxidizing aerobic enrichment culture in which mycobacteria (phylum *Actinobacteria*) were numerically dominant [∼ 95% of 4′,6-diamidino-2-phenylindole (DAPI)-stained cells]; and (vi) a micro-oxic methane-oxidizing enrichment culture, dominated by a *Methylococcaceae* species (∼ 97% of DAPI-stained cells; Tavormina *et al*., in review).

**Fig 2:**
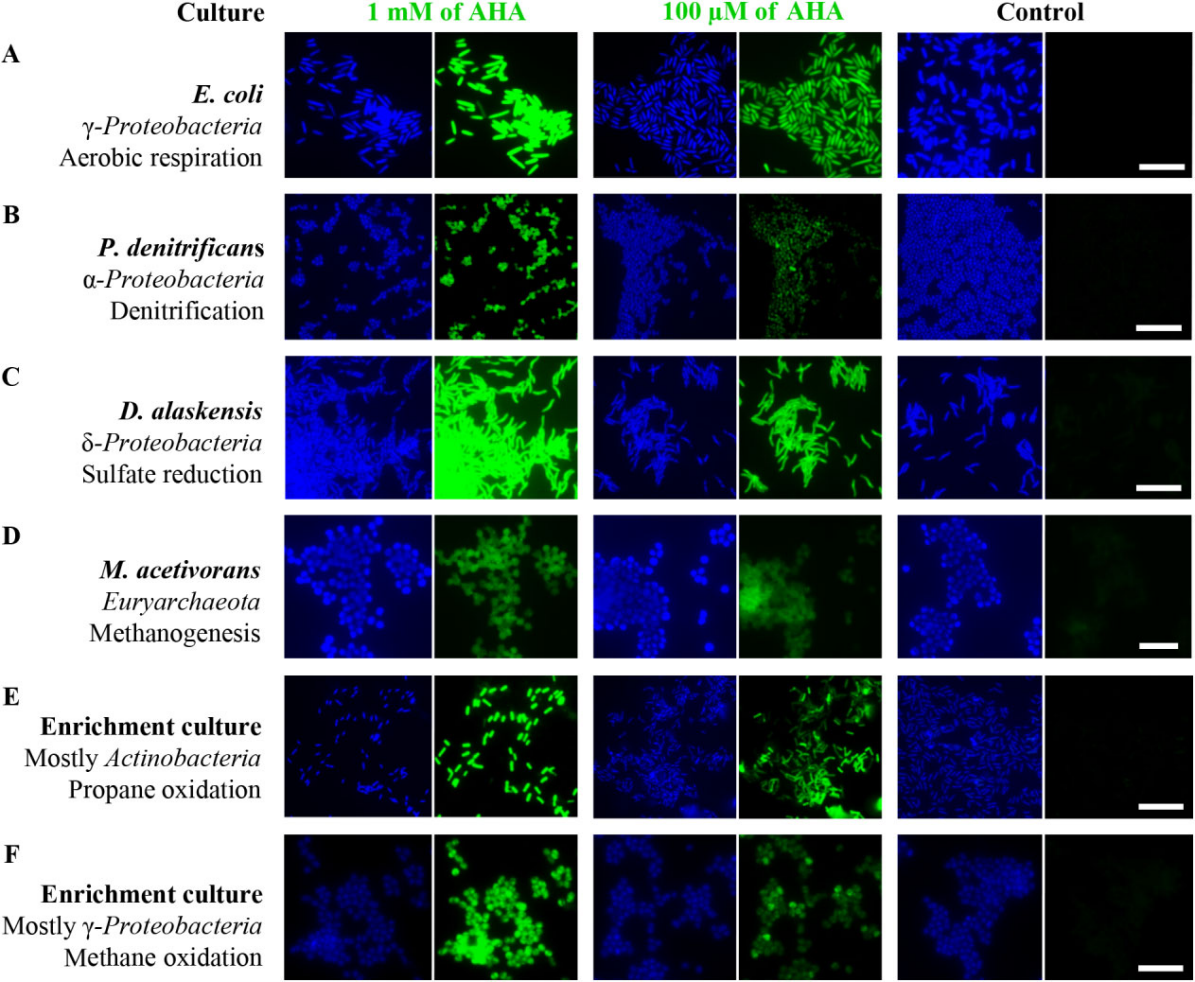
Uptake and incorporation of AHA is independent of the physiological or phylogenetic background of the target organism. Different pure and enrichment cultures were incubated in the presence or absence of AHA. BONCAT signals (green) were taken at identical exposure times for individual series (i.e. 0.1 and 1 mM AHA plus control). Note that incubation conditions were different for the individual cultures, cells have contrasting levels of background fluorescence, and that different labelling strategies were used. Together, these issues limit the value of a direct comparison of signal intensities between different cultures. DAPI-staining is shown in blue. All scale bars equal 10 μm and apply to each set of images respectively. A–D. BONCAT-labelling of four bacterial and archaeal pure cultures. E–F. BONCAT-labelling of propane- and methane-oxidizing enrichment cultures.

Cultures were incubated in the presence or absence of AHA (100 μM or 1 mM) and cells that incorporated AHA during the incubation were identified via click chemistry using fluorophores Carboxyrhodamine 110 Alkyne and DBCO-PEG_4_-Carboxyrhodamine 110 (Fig. 2). BONCAT-labelling of an *E. coli* culture that had been incubated in the presence of AHA (1 mM) and the protein synthesis inhibitor chloramphenicol (Camp) did not yield fluorescently labelled cells (data not shown). All incubations were performed for ≤ 1 generation of the respective culture (as assessed by OD_600_) to minimize excessive substitution of Met with AHA. Substantial replacement with a non-canonical amino acid such as AHA could interfere with the efficiency of the cellular machinery over time. Notably, with such short incubation times, AHA was not observed to influence the growth rate of the cultures and fluorescently labelled inclusion bodies, which can represent aggregations of misfolded proteins (Fahnert *et al.*, 2004), were never detected within the cells. For cultures that had been incubated in the presence of AHA, generally > 97% of all DAPI-stained cells were fluorescently labelled (Fig. 2). The inability to stain the remaining cells could be due to one or a combination of the following reasons: (i) BONCAT-negative cells were not metabolically active during the incubation or did not express the respective uptake machinery (which is currently unknown for AHA); or (ii) the number of incorporated AHA molecules was insufficient to be detected via conventional fluorescence microscopy. In our parallel 16S rRNA-targeted FISH experiments (see below) essentially all cells (≥ 99%) within the individual cultures were FISH-labelled. We did not observe a difference in FISH signal intensity of BONCAT-negative as compared with positive cells. This is consistent with the finding that rRNA FISH often is not a reliable tracer of metabolic activity in microorganisms (e.g. Wagner *et al.*, 1995; Binder and Liu, 1998; Morgenroth *et al.*, 2000; Schmid *et al.*, 2001; Bollmann *et al.*, 2005). It should, however, be noted that the majority (visual estimate) of BONCAT-negative cells demonstrated weak DAPI signals, which suggests a lower DNA content as compared with intensively DAPI-stained cells (Fig. 2). Considering that fluorescently labelled oligonucleotide probes have molecular weights equal to or larger than the clickable dyes tested here, ineffective cell permeabilization is not likely to be the cause of the inability to label all cells using BONCAT.

In addition, we performed labelling experiments using Carboxyrhodamine 110 Alkyne using *E. coli* and *D. alaskensis* cultures that had been incubated in the presence of 10 μM AHA. We observed comparable labelling efficiencies (> 97% of DAPI-staining cells; data not shown), demonstrating that under defined conditions (no Met in the medium) AHA concentrations in the range of several μM are sufficient to effectively label bacterial proteins.

We further attempted to fluorescently label AHA-containing proteins in living microorganisms, here referred to as ‘liveBONCAT’. Our preliminary results, which are summarized in the supplementary online information (Supporting Information Fig. S2), suggest that it may be possible to fluorescently label microbial cells *in vivo*, although the existing protocol requires further testing and optimization to enhance the percentage of cell labelling.

### In gel detection of newly synthesized proteins

**Fig 3:**
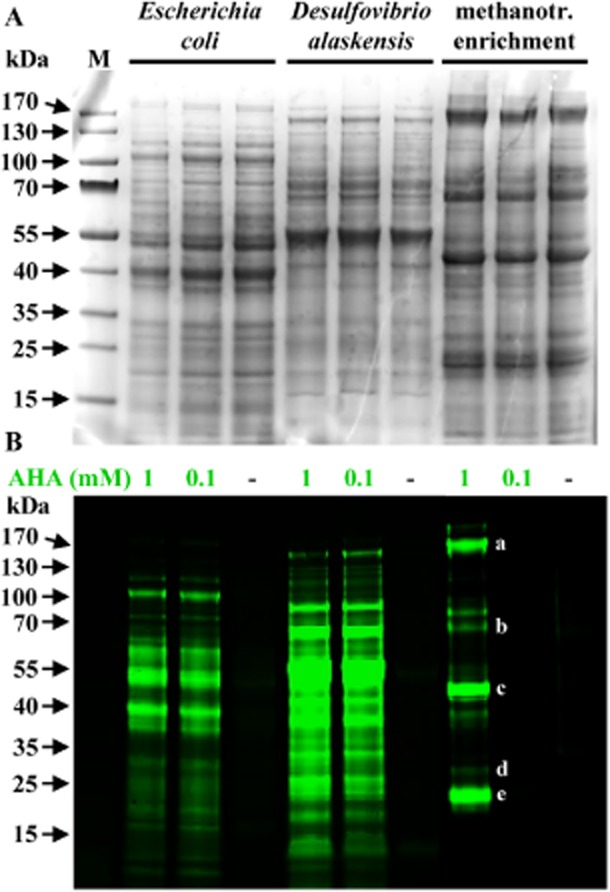
AHA does not interfere with the cellular machinery. Visualization of new proteins from cultures of *E. coli*, *Desulfovibrio alaskensis* and a methanotrophic enrichment. A. Coomassie-stained protein band patterns of cultures that had been incubated in the absence (−) or presence of AHA are indistinguishable from each other, demonstrating that AHA does not interfere with the translational machinery. B. Newly made proteins in the same gel are identified via BONCAT. Please note that the incubation time for the methanotrophic culture exposed to 100 μM AHA was too short to yield new proteins in amounts high enough to be detectable via *in-gel* fluorescence. At the individual cell level, AHA uptake can, however, be easily demonstrated (Fig. 2F). Some of the most intensely labelled bands were cut from the gel and analyzed via mass spectrometry. The 20 most abundant proteins from the excised bands included: (a) the two large subunits of RNA-polymerase (150.4 and 155.4 kDa); (b) a hypothetical protein (67.8 kDa) as well as two homologs of the large subunits of methanol dehydrogenase (66.6 and 68.3 kDa); (c) PmoB, 45.6 kDa; (d) PmoA (28.4 kDa) and PmoC (29.1 kDa); (e) a formaldehyde activating enzyme (17.8 kDa), superoxide dismutase (21.1 kDa), and D-arabino-3-hexulose 6-phosphate formaldehyde lyase (21.8 kDa). Letters a–e denote the bands consistent with the molecular weights of these proteins. kDa, kiloDalton; M, marker.

**Fig 4:**
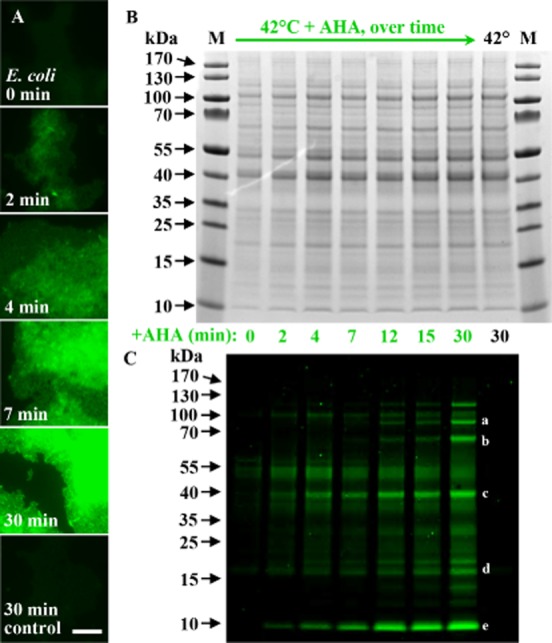
The high sensitivity of BONCAT allows detection of newly synthesized proteins after only minutes of incubation. A. Fluorescence labelling of heat-shocked (42°C) *E. coli* cells grown in the presence of AHA increases over time. Already after 2 min of incubation, equivalent to about 2% of *E. coli*'s generation time under the conditions used, labelled cells can be visualized. B, C. While no differences in the relative amounts of proteins can be observed via Coomassie-staining, the fluorescent tag conjugated to newly made proteins reveals that certain proteins are preferentially amplified with time. C. Some of the most intensely fluorescently labelled bands were cut from the gel and analyzed via mass spectrometry. The obtained proteins included: (a) DNA gyrase subunits A and B as well as chaperone protein ClpB (97.0, 89.9 and 95.6 kDa respectively); (b) chaperonin GroL, a heat-inducible Lysine-tRNA ligase and chaperone DnaK (57.3, 57.8 and 69.1 kDa respectively); (c) outer membrane protein A (37.2 kDa); (d) chaperone proteins Skp (17.7 kDa) and YajL (20.8 kDa); (e) nine ribosomal proteins of low molecular mass (≤ 10 kDa) and the highest abundant protein in our dataset, major outer membrane lipoprotein Lpp (8.3 kDa). Letters a–e indicate bands consistent with the molecular weights of these proteins. Control refers to 30 min incubation at 42°C in the absence of AHA. Fluorescent signals were recorded at identical exposure times. Scale bar equals 10 μm and applies to all images. kDa, kiloDalton; M, marker.

To test whether added AHA was directly incorporated into newly made proteins and whether this non-canonical amino acid alters the translational activity (or results in protein degradation), proteins were extracted from *E. coli* (3 h of incubation at 32°C, i.e. ∼ 0.67 generations), *D. alaskensis* (3.5 h, i.e. ∼ 0.50 generations) and the methanotroph enrichment culture (26 h of incubation, equal to ∼ 0.59 generations as judged by change of OD_600_) amended with AHA (0.1–1 mM). Extracted proteins were conjugated with dye DBCO-PEG_4_-Carboxyrhodamine 110 using strain-promoted click chemistry and analyzed by gel electrophoresis. No difference in Coomassie-stained protein bands were observed between cultures that had been incubated in the presence or in the absence of AHA (examples are shown in Fig. 3A). This is in accordance with our growth rate analyses as well as recent studies that demonstrated using quantitative proteomics that addition of AHA does not lead to protein identification artefacts in *E. coli* or different human cell lines (Eichelbaum *et al.*, 2012; Bagert *et al.*, 2014). Three independent AHA incubation experiments with *E. coli*, *D. alaskensis* and a gammaproteobacterial methanotrophic enrichment culture all demonstrated the incorporation of AHA into a diverse range of proteins (Fig. 3B). For the methanotrophic enrichment, there was a discrepancy in the apparent detection limit when comparing in gel fluorescence labelling of proteins and whole-cell detection via click chemistry. While both 100 μM and 1 mM AHA incubations yielded fluorescently labelled whole cells (Fig. 2F), fluorescent protein bands were not detected in the incubation with 100 μM AHA and only a small number of fluorescent protein bands was detected in the 1 mM AHA treatment. These results suggest that single-cell fluorescence labelling of microbial cells has a lower detection limit than in gel fluorescence. Consistent with the low molecular weight of the dye (0.88 kDa) we did not observe differences between the migrational patterns of unlabelled as compared with labelled proteins (Figs. 3A and 4B).

Proteomic analysis was conducted on excised fluorescent bands from the *Methylococcaceae* sp. WF1 enrichment shown in Figure 3B (*n* = 5 fluorescently labelled bands). Consistent with the numerical dominance of *Methylococcaceae* in the enrichment culture, the calculated molecular weights of the most intensely labelled bands recovered from the 1 mM AHA incubation were consistent with enzymes expected to be highly expressed in aerobic methanotrophs, e.g. the subunits of particulate methane monooxygenase: PmoA (28.4 kDa), PmoB (45.6 kDa) and PmoC (29.1 kDa; Trotsenko and Murrell, 2008). The presence of PmoABC in the excised bands was additionally confirmed by mass spectrometry. Pmo subunits were among the 20 most abundant proteins in the data set, along with two homologs of the large subunit of methanol dehydrogenase (66.6 and 68.3 kDa), and the large subunits of DNA-directed RNA polymerase (150.4 and 155.4 kDa; Fig. 3B; Supporting Information Table S1A).

Although the sensitivity of these experiments is limited by the low-resolution power of one-dimensional protein gels, these results demonstrate that AHA is incorporated into a diverse range of cellular proteins and does not alter the synthesis or degradation of proteins. In contrast to Coomassie-staining, which is only able to visualize highly abundant proteins, click chemistry-based dye conjugation of AHA-containing proteins is able to distinguish highly expressed proteins from low turnover proteins with long life times.

### Sensitivity of BONCAT

The sensitivity of BONCAT is influenced by several factors. Among the most important and experimentally testable are the Met content of the targeted proteins as well as the growth rate and translational activity of the cell. The average number of Met per candidate protein for the microorganisms studied here ranges from 7.0 (draft genome of *Methylococcaceae* sp. WF1) to 9.6 (*D. alaskensis*). Every protein encoded in the five genomes has at least one Met, and 10.0% (*Methylococcaceae* sp. WF1) to 18.9% (*D. alaskensis*) of all proteins have at least 15 Met in their predicted sequence (Supporting Information Fig. S3). Presumably, labelling efficiency is mostly dependent on the intracellular ratio of AHA/Met and the selectivity of the respective methionyl-tRNA synthetase. Thus, while perfect labelling efficiency is not expected, the large number of Met residues across the proteomes indicates the theoretical potential to label and detect every protein that is newly synthesized by these microorganisms.

Time course experiments with *E. coli* cultures were used to determine the minimum time required for detection of AHA-labelled proteins by BONCAT. Here, a heat-shock experiment with *E. coli* grown in the presence or absence of 1 mM AHA was performed, and then intact cells and protein extracts were analyzed after 2 to 30 min of incubation. Within 2 min of incubation, equivalent to ∼ 2% of *E. coli's* generation time under the experimental conditions, fluorescently labelled proteins were detected in gels as well as on the cell level (Fig. 4). Furthermore, while no changes in *E. coli's* total proteome were observed in Coomassie-stained protein gels, the visualization of newly made proteins via click chemistry revealed a high turnover rate for specific proteins (Fig. 4B,C). We performed proteomic analysis on several excised fluorescent bands (Fig. 4C). In total, 250 proteins were retrieved (Supporting Information Table S1B), which included several heat-shock proteins, specifically DNA gyrase subunits A and B (97.0 and 89.9 kDa), a heat-inducible Lysine-tRNA ligase (57.8 kDa), as well as chaperone proteins ClpB (95.6 kDa), GroL (57.3 kDa), DnaK (69.1 kDa), Skp (17.7 kDa), and YajL (20.8 kDa). Other highly abundant proteins contained within the excised fluorescent bands included outer membrane protein A (37.2 kDa) as well as 17 proteins of low molecular mass (≤ 10 kDa; including nine ribosomal proteins), as well as the highest abundant protein in the obtained data set, major outer membrane lipoprotein Lpp (8.3 kDa). Together, these observations demonstrate the potential of BONCAT to study rapid proteomic adaptation of fast growing microorganisms in response to environmental stimuli.

### BONCAT-FISH: correlating translational activity with microbial identity

**Fig 5:**
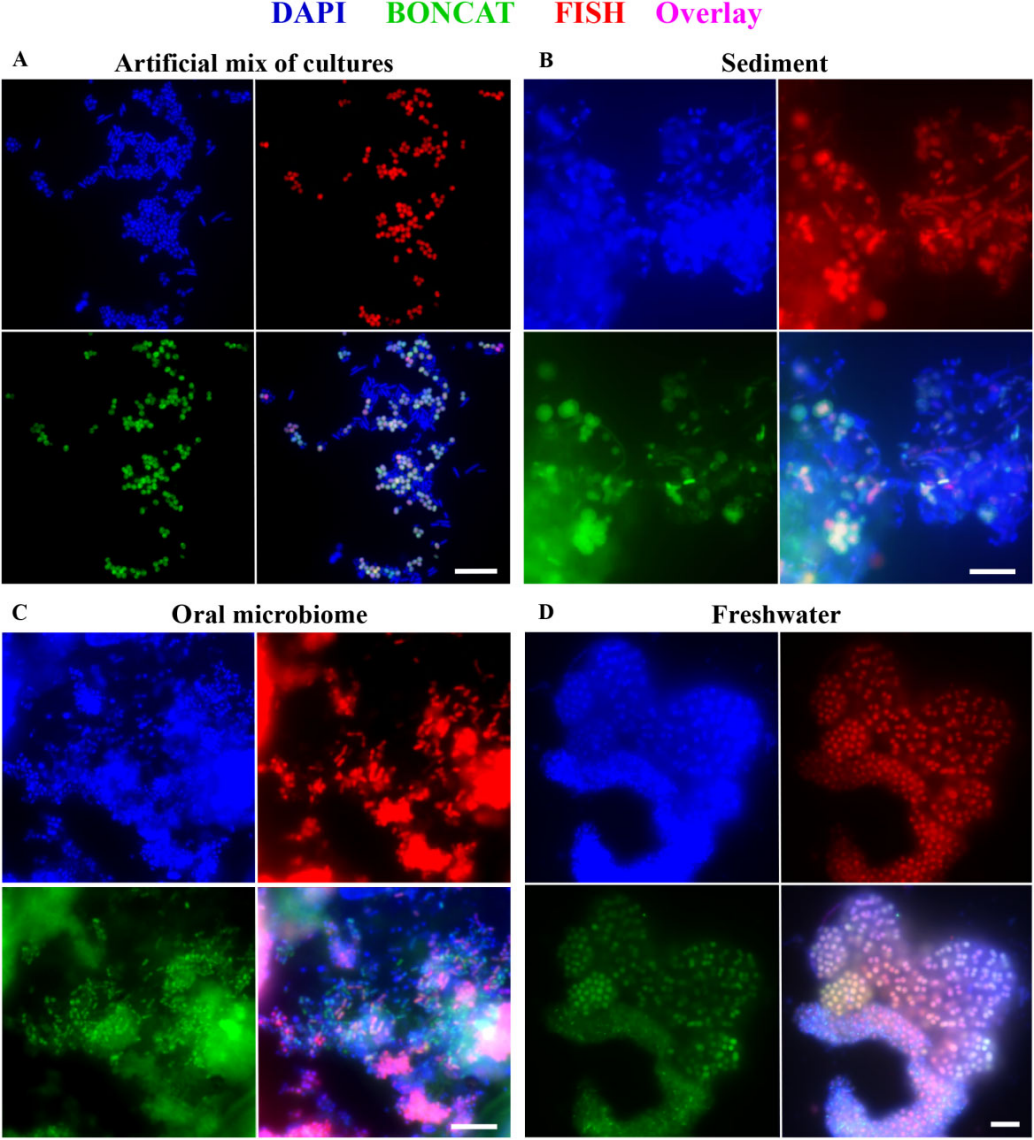
Visualization of newly synthesized proteins via BONCAT (green) in combination with rRNA-targeted FISH (red). A. An artificial mix of pure and enrichment cultures. The only microbe that had been incubated in the presence of AHA, a gamma proteobacterial methanotroph (*Methylococcaceae* sp. WF1), is identified via a species-specific FISH probe (MetI-444), demonstrating the feasibility of correlating translational activity with microbial identity. For details and a Cy5-probe image see Supporting Information Fig. S4. B–D. Many bacteria, identified by the general EUB338I-III FISH probe mix (B,C) or probe Gam42a (D), specific for gammaproteobacteria, are BONCAT-labelled, demonstrating *in situ* translational activity during time of incubation. Exposure times for click or FISH signals were identical for each sample series (i.e. AHA plus two controls), respectively. DAPI staining is shown in blue. For controls, see Supporting Information Figs. S4 and S6. All scale bars equal 10 μm.

One of the most powerful characteristics of BONCAT is its potential to study the *in situ* activity of microbes in complex samples on the level of individual cells. To correlate translational activity with taxonomic identity, BONCAT was combined with 16S and 23S rRNA-targeted FISH. Defined mixtures of pure cultures and enrichments of bacteria and archaea, in which one of the cultures had been incubated with AHA prior to mixing with other cultures, were used to develop the BONCAT-FISH protocol (Fig. 1C). Immobilized cells from the formaldehyde (FA) fixed mixed cultures were fluorescently labelled via click chemistry and subsequently hybridized using general and species-specific oligonucleotide probes and nonsense control probes (Fig. 5A and Supporting Information Fig. S4). A comparison of mixed cultures that had been analyzed by labelling of new proteins and FISH in contrast to microbial cells stained only by protein-targeted click chemistry revealed comparable fluorescence intensities (data not shown). By combining BONCAT with FISH, we were able to detect active protein synthesis by the novel *Methylococcaceae* sp. WF1, which was identified using a specific FISH probe (MetI-444) within a mixture of other bacteria and archaea (Fig. 5A). The successful combination of FISH and BONCAT demonstrates the possibility to directly link species-specific identification of microbes with detection of their translational activity for individual cells using conventional fluorescence microscopy.

### Using BONCAT to assess the physiological potential of microorganisms in a mixed community: experiments with a methanotrophic enrichment culture

**Fig 6:**
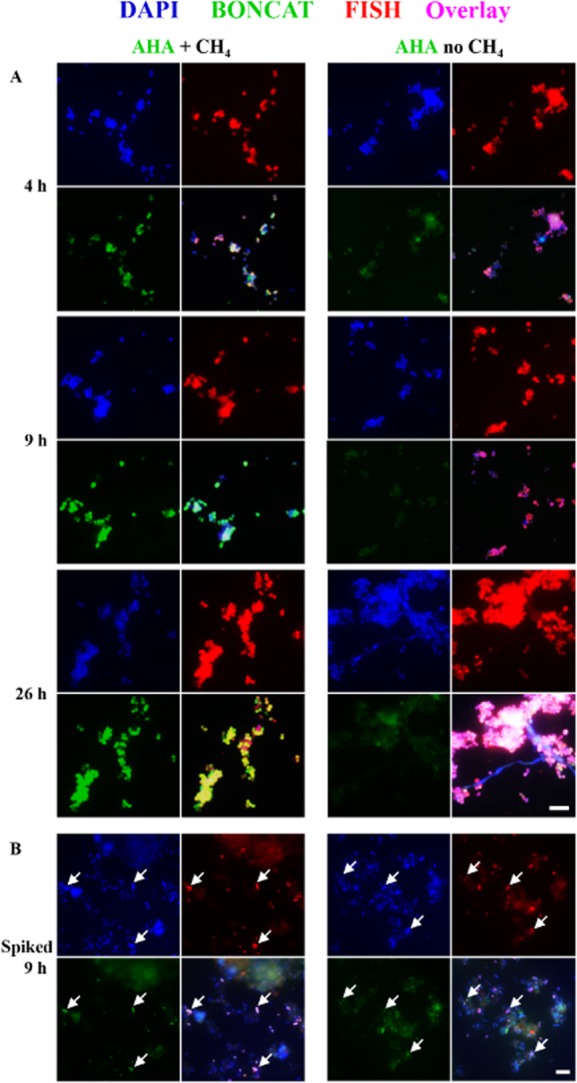
Comparative BONCAT analyses of a methanotrophic enrichment culture in the absence and presence of methane. A. Click chemistry-mediated detection of AHA incorporation (green) reveals that a gammaproteobacterium (identified by FISH probe MetI-444; red) is highly active in the presence but not in the absence of methane. B. To test whether AHA-labelled WF1 cells (examples are pointed out by arrows) would be detectable in a complex samples, an aliquot of the culture was spiked into methane seep sediment and analyzed via BONCAT. Exposure settings for recording of BONCAT signals were identical for each image set (i.e. A, B). For images taking at different settings and controls, see Supporting Information Fig. S5. Scale bars equal 10 μm and apply to all images of the respective set.

The ability to rapidly screen the anabolic activity of specific microorganisms in the environment using BONCAT-FISH offers a mechanism for assessing the activity of FISH-identified environmental microorganisms in response to substrate addition or physical manipulation in environmental samples. To demonstrate the ability to use BONCAT-FISH for discerning the physiological response of microbes under different conditions, we tested the effect of methane addition on an aerobic methanotrophic enrichment culture (WF1) originating from deep-sea sediments. This enrichment was dominated by a *Methylococcaceae*-related gammaproteobacterium based on FISH analyses using the methanotroph-specific probe MetI-444. Aliquots of the WF1 enrichment were incubated with 1 mM AHA in the presence or absence of methane for 26 h and then analyzed by BONCAT-FISH. FISH-identified *Methylococcaceae* cells showed strong BONCAT fluorescence signal (i.e. high translational activity) that increased over time in incubations with methane + oxygen, whereas a BONCAT signal was not detected in FISH-stained WF1 cells incubated without methane (Fig. 6A and Supporting Information Fig. S5). This comparative incubation experiment using BONCAT-FISH, together with recently obtained genomic data from this deep-sea *Methylococcaceae* strain (Tavormina *et al*., in review) and mass spectrometric analyses of its proteins (herein), provides clear evidence for the methanotrophic nature of this bacterium. To further test whether AHA-containing WF1 cells can be distinguished from other sediment microorganisms, we spiked an aliquot of the AHA-labelled *Methylococcaeae* culture (sample after 9 h of incubation) into a marine sediment sample and used Cu(I)-catalyzed click chemistry with a Carboxyrhodamine 110 Alkyne dye to screen the sample. As demonstrated in Fig. 6B, active *Methylococcaceae* cells can be clearly distinguished from other sediment-dwelling microbes via BONCAT. These results demonstrate the potential of using BONCAT-FISH for testing the physiology of microbes in complex environmental samples and highlight its use as a rapid and inexpensive screening tool for studying the response of cultured and uncultured microbes towards environmental stimuli.

### Comparison of single-cell metabolic activity proxies: BONCAT versus nanoSIMS ^15^N/^14^N analysis

Over the past 15 years, there have been a number of methods using whole-cell isotope labelling combined with FISH to measure translational activity and growth of individual microorganisms in complex environmental samples. These techniques rely on the incorporation of radiolabelled (^3^H, ^14^C or ^35^S) amino acids into cellular proteins using MAR or stable isotope-labelled ammonia or amino acids followed by microRaman or SIMS analyses. While these isotopic methods are gaining increasing use in microbial ecological studies, there are challenges impacting their general application by microbiologists: (i) assimilated ammonia can be used for other cellular functions in addition to translation, which may complicate data interpretation; (ii) limited access to radioactivity-certified laboratories or expensive microRaman or nanoSIMS instruments; and (iii) with SIMS and nanoSIMS analyses, considering the trade-offs between a high-sensitivity and precise measurement with relatively low sample throughput. From this perspective, BONCAT offers some advantages in accessibility and cost as compared with isotopic approaches. BONCAT uses commercially available and relatively inexpensive reagents and requires only standard molecular biological equipment (e.g. an epifluorescence microscope), offering a fast, culture-independent and cost-effective approach for direct analysis of anabolic activity in microorganisms in the environment.

**Fig 7:**
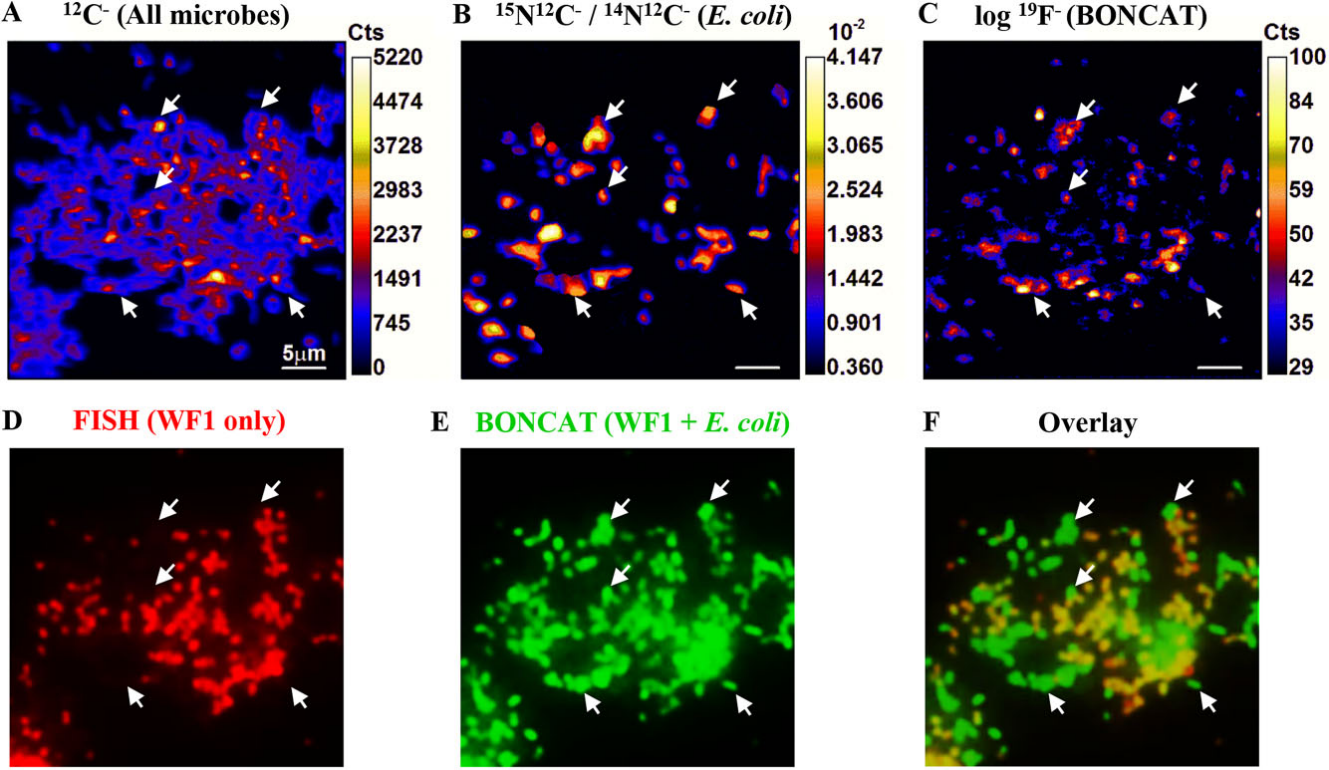
Comparison of AHA labelling of newly synthesized proteins with cellular ^15^N-uptake. An artificial mix of several cultures was analyzed via BONCAT, FISH and nanoSIMS. *Escherichia coli* had been incubated in the presence of both ^15^NH_4_Cl and AHA, while a methanotroph was exposed to AHA only. Other microbes had been grown in the absence of AHA or ^15^NH_4_Cl. A species-specific FISH probe (MetI-444) is used to localize the methanotroph WF1 (D; red), while both WF1 as well as *E. coli* are fluorescently labelled by a fluorine-containing alkyne-dye (E; green). While all *E. coli* cells (examples are pointed out by arrows) are ^15^N- and ^19^F-labelled (B, C), the ^19^F-signal of methanotroph cells is indistinguishable from cells that had not been incubated in the presence of AHA (C). A second halogen-containing dye (see Supporting Information Fig. S1) could not be used due to problems removing unbound dye (see main text). Scale bars equal 5 μm. Abbreviations: Cts, counts; WF1, cells of *Methylococcaceae* sp. WF1. A–C. Elemental and isotopic mapping of an artificial mix of cultures via nanoSIMS. D–F. Correspondent FISH and BONCAT images of the same field of view.

To directly compare BONCAT-FISH and isotopic labelling methods by nanoSIMS, an artificial mixture of cultured microorganisms that had been grown in the presence of either AHA or AHA + ^15^NH_4_^+^ was prepared and then analyzed by both fluorescence microscopy and nanoSIMS. *Escherichia coli* cultures grown in the presence of 1.87 mM ^15^NH_4_Cl (i.e. 9% of total ^14+15^NH_4_^+^ pool) and 1 mM AHA were mixed with a methanotrophic enrichment culture (WF1) that had been incubated with 1 mM AHA and three unlabelled pure cultures of archaea and bacteria in roughly equal cell abundances. Cu(I)-catalyzed click chemistry using the ^19^F-containing dye Oregon Green 488 alkyne (Supporting Information Fig. S1A) followed by FISH was performed on the mixed culture and cells were imaged via fluorescence microscopy and by nanoSIMS to measure ^15^N enrichment and ^19^F content for each cell (Fig. 7). Using a specific FISH probe (MetI-444), we identified BONCAT-labelled cells of methanotroph WF1 in the mixed culture by epifluorescence (Fig. 7D), while *E. coli* cells were identified by ^15^N enrichment in the ^15^N/^14^N ratio images acquired by nanoSIMS (Fig. 7B). Both WF1 and *E. coli* exhibited strong BONCAT signals after incubation with AHA, with *E. coli* exhibiting slightly stronger relative fluorescence intensities per cell (Fig. 7E). All ^15^N-containing *E. coli* cells were fluorescently labelled (and *vice versa*, all BONCAT-positive *E. coli* cells had elevated ^15^N levels), and enriched in ^19^F (average ^19^F/^12^C = 0.0341; *n* = 35 regions of interest, ROI) as compared with non-AHA-labelled cells of different taxonomy (average ^19^F/^12^C = 0.0185; *n* = 15 ROI). However, WF1 methanotroph cells (average ^19^F/^12^C = 0.0177; *n* = 20 ROI; Fig. 7C) were indistinguishable from these control cells (*t*-test *P*-value = 0.359). This observation has two possible explanations: (i) *E. coli* cells had incorporated higher amounts of AHA and thus were more intensely labelled with the ^19^F-containing fluorescent dye. This possibility is supported by the fact that the *E. coli* culture was grown for ∼ 0.89 generations (1.4 h of incubation time; final OD ∼ 0.23) in the presence of AHA, while the WF1 enrichment had been exposed to AHA only for ∼ 0.59 generations (25.7 h; final OD ∼ 0.17). However, there was a smaller difference in relative fluorescence signal intensity between *E. coli* and methanotroph WF1 cells compared with cellular ^19^F enrichment by nanoSIMS (Fig. 7E versus 7C). This discrepancy may be related to the difference in detection method and subtle differences in the cellular properties of the WF1 and *E. coli* cells. While the epifluorescence image represents an integral of the click signal of the whole cell, the nanoSIMS-based ^19^F measurement is a destructive sampling method, where secondary ions are sputtered from the cell, quantified and then averaged over a Z-stack that may represent a few atomic layers to nanometers depending on the depth of pre-sputtering and analysis time. Independent of the discrepancy in fluorescence intensity and ^19^F-labelling, the results from these AHA incubation experiments are largely consistent with cellular ^15^NH_3_ assimilation as analyzed by nanoSIMS, demonstrating the high potential of BONCAT for studying translationally active cells by means of fluorescence microscopy.

### Visualizing active microbes in environmental samples using BONCAT-FISH

AHA incubation experiments and BONCAT-FISH were also applied to samples collected from a range of environments, including the oral microbiome (tongue scraping and saliva), pond water and anoxic sediment, to study taxonomically and physiologically diverse microorganisms within a range of sample types. These samples were selected to serve as a testing ground for the application of BONCAT-FISH with natural samples, and were incubated for several hours (4, 6 and 7.5 h for freshwater, oral and sediment samples respectively) in the presence or absence of AHA (1 mM). FA-fixed cells from each of the sample incubations were analyzed via click chemistry followed by FISH using both general (e.g. EUB338mix, Gam42a) as well as species-specific (MetI-444) oligonucleotide probes (Fig. 5B–D and Supporting Information Fig. S6).

In all samples, microbial cells with diverse morphologies were observed to be translationally active during the incubation period, which suggests a general applicability of BONCAT for microbial ecology studies. The high abundance of conspicuous aggregations of purple sulfur bacteria in the freshwater sample during time of sampling and the subsequent FISH identification of the majority of cells in dense cell clusters as gammaproteobacteria (probe Gam42a), make it likely that these BONCAT-labelled cells are members of the order *Chromatiales* (Fig. 5D). There were also observable differences in the anabolic activity of microorganisms between habitats. While the majority of cells that hybridized with the general bacterial probe (EUB338mix) in the oral and freshwater samples were also labelled by BONCAT (Fig. 5C and D), only a small proportion of the FISH-identified bacteria in the sediment sample were BONCAT-labelled (Fig. 5B). Unfortunately, absolute quantification of labelling efficiency was not possible because of the compact and densely packed nature of cells in all three samples. The most probable explanation for the inability to label the majority of sediment-affiliated cells is that (i) most sediment microbes did not synthesize proteins in high enough amounts for fluorescence microscopic detection during the incubation period. However, it cannot be excluded that (ii) some microbes did not take up AHA into their cells or proteins, possibly because of the absence of appropriate transporters, the high selectivity of their methionyl-tRNA-synthetases, or a high Met/AHA ratio in the cytoplasm. Arguably, the most crucial limitation of BONCAT is its dependence on an uptake mechanism. However, this is not specific to our approach, because every metabolic labelling technique is constrained by its need for an uptake system for the respective compound, be that ammonia, an amino acid, or a redox dye.

### Stability of AHA

AHA is stable under all physiological and environmental conditions with the exception of high concentrations of sulfide and alkaline pH. Under such conditions, bisulfide (HS^-^) reduces organic azides selectively to the corresponding amines (Adachi *et al.*, 1977). To test the extent of reduction of AHA by HS^-^, we performed abiotic, anoxic incubations of 1 mM AHA in the absence or presence of sulfide (H_2_S plus HS^−^ equaling 1, 2 or 10 mM) and analyzed AHA and its reduction product, *L*-2,4-diaminobutyric acid, via nuclear magnetic resonance (NMR) spectroscopy at regular time intervals (Supporting Information Fig. S7). We found that at pH 7.0, identical to the pH of our sediment sample, after 8 days sulfide has no measurable effect on AHA (data not shown). At pH 8.0, the effect of sulfide increases with its concentration, with ≥ 10 mM being enough to reduce > 30% AHA within 40 h and concentrations ≤ 1 mM having only a minor effect (reduction of ∼ 5% AHA; Supporting Information Fig. S7). Considering that (i) typical sulfide concentrations in the sediment studied here are around the detection limit of our method (i.e. 60 μM; determined via cline assay), (ii) the pH of the sample was ≤ 7, and (iii) incubation times were substantially shorter than in our abiotic reactions (7.5 h versus 140 h), we conclude that during our freshwater and sediment incubations HS^-^ had no significant effect on the concentration of AHA.

While not the focus of this study, some habitats inhabited by microbes with particularly slow growth rates exhibit conditions that could restrict the use of AHA over the course of long incubation times (i.e. several days to months). Examples of such systems include sulfidic deep-sea methane seeps in which inter-domain consortia, with doubling times of ∼ 3 months, catalyze the anaerobic oxidation of methane (reviewed by Knittel and Boetius, 2009). If such a system is to be studied we recommend either adding AHA at regular intervals during the incubation, or, better, using alternative surrogate amino acids (discussed below).

To test the stability of AHA at elevated temperatures, AHA (10 mM in water) was incubated at 80 ± 3°C and analyzed via NMR spectroscopy after 19.5 h of incubation. We found that at pH 5.0 and 7.1, high temperature has no measurable effect on AHA (data not shown), allowing for the study of protein turnover in (hyper)thermophilic microorganisms.

### Considerations for BONCAT-FISH experiments of environmental samples

The activation rates of enzymes, specificities of transporters, etc. for bioorthogonal compounds as compared with their canonical counterparts (AHA versus Met) are likely lower. To our knowledge, the only enzyme that has been comparatively studied in this regard is the methionyl-tRNA synthetase of *E. coli*, where the activation rate for AHA is ∼ 0.25% of that for Met (Kiick *et al.*, 2002). Thus, in respect to this enzyme a ‘total’ concentration of 100–1000 μM AHA in a sample translates to 0.25–2.5 μM of ‘bio-available’ amino acid. While these concentrations are still 100–1000 times higher than standing concentrations of amino acids in highly oligotrophic systems (e.g. open-ocean waters), the very low competition of *in situ* Met with AHA in such habitats would presumably facilitate the use of lower concentrations of AHA.

Given the complex structure, biochemistry and molecular regulation of gene expression in microbes, it is preferable to keep incubation times at a minimum to reduce the risk of system disturbance. For the environmental samples examined here, incubation times were comparable with or shorter than what is typically used in MAR- and SIMS-based experiments with incorporation of ammonia or amino acids (e.g. Ouverney and Fuhrman, 2000; Sintes and Herndl, 2006; Teira *et al.*, 2006; Behrens *et al.*, 2008; Finzi-Hart *et al.*, 2009; Orphan *et al.*, 2009; Pernice *et al.*, 2012), and much shorter than in typical microRaman experiments (e.g. Huang *et al.*, 2004; Huang *et al.*, 2007; Haider *et al.*, 2010). If longer incubation times are required, e.g. due to the study of ultra-oligotrophic systems or slow-growing microorganisms (e.g. Dekas *et al.*, 2009; Morono *et al.*, 2011), additional approaches that independently test for community succession or habitat disturbance should be conducted. Examples include analysis of 16S rRNA gene diversity over time, FISH, as well as geochemical and rate analyses to assess the activity of the microbes of interest.

In summary, AHA-based BONCAT-FISH is a broadly applicable technique, with the exception of samples harbouring high concentrations of sulfide in combination with high pH (e.g. sulfidic sediments) or systems characterized by high concentrations of Met. Because Met is a better substrate than AHA with respect to protein synthesis (Kiick *et al.*, 2002), microbes living in extremely high nutrient environments, e.g. animal or human guts, will probably require modified BONCAT protocols. This idea is supported by the finding that if nutrient-rich media (Lysogeny broth, LB) were used, the high concentration of Met in these media reduced the incorporation of AHA into newly made *E. coli* proteins to levels below our limits of microscopic detection (data not shown).

### Alternative clickable amino acid surrogates

Besides AHA, other bioorthogonal amino acids amenable to click chemistry have been described, including, most importantly, the Met surrogates homopropargylglycine (HPG) and azidonorleucine, as well as several analogs of pyrrolysine (Kiick *et al.*, 2002; Fekner *et al.*, 2009; Ngo and Tirrell, 2011). HPG previously has been used for the study of newly synthesized proteins in mammalian fibroblasts (Beatty *et al.*, 2010) and neurons (Dieterich *et al.*, 2010). Some alternatives, however, have limitations compared with the more robust and generally applicable AHA. For example, HPG has a substantially lower activation rate than AHA, while azidoalanine does not support protein synthesis in *E. coli* (Kiick *et al.*, 2002). However, it has to be considered that activation rates might potentially substantially differ in phylogenetically distant and/or physiologically distinct microbes. Surrogates of unusual amino acids like pyrrolysine, for which the phylogenetic range is restricted to some groups of methanogens and deltaproteobacteria (Hao *et al.*, 2002; Zhang and Gladyshev, 2007), may also have utility in targeted studies.

### Outlook: combining BONCAT with established techniques

The successful application of BONCAT-FISH in natural ecosystems demonstrates the feasibility of correlating single-cell translational activity with phylogenetic identity *in situ*. In contrast to current methods used for measuring single-cell activity in microbial ecology, BONCAT enables the direct visualization of newly synthesized proteins via fluorescence microscopy, a technique commonly available in molecular biological laboratories. This method is simple, fast and comparatively high throughput using commercially available, inexpensive reagents. As azide-containing molecules are rare in the environment, the potential for nonspecific reactions is minimized. The chemistry of the click reaction is well understood, exhibits high specificity and is easily performed in the presence of a complex inorganic or organic matrix. Combined with rRNA-targeted FISH, BONCAT holds exciting prospects for the study of the spatio-temporal dynamics, ecophysiology and *in situ* anabolic activity of environmental microbes.

While not directly tested in this study, there are a number of promising future applications of BONCAT. For example, combining this technique with fluorescence-activated cell sorting would enable physical separation of translationally active cells, an approach analogous to the respiration response imaging method using redox sensor green (Kalyuzhnaya *et al.*, 2008; Konopka *et al.*, 2011). Quantification of the relationship between spatial organization and anabolic activity within structured microbial communities such as microbial mats, biofilms or consortia is also possible. Such a combination of techniques has the potential to grant us access to the genomes and physiologies of unidentified species and is independent of their respective numerical abundance in the environment.

The dynamic BONCAT fluorescent labelling of cells in response to substrate availability, demonstrated in this study with methanotrophs maintained in the presence or absence of methane, may also have utility for selective cultivation of microorganisms using liveBONCAT. Our preliminary results suggest that, with some optimization, fluorescence-labelling of living microorganisms after AHA incubation is possible. Extended to natural ecosystems, liveBONCAT could be used as a screening tool to study the reaction of uncultured microbes to the addition of potential substrates. Candidate cells that were translationally active in the presence of a compound of interest could be physically separated and subjected to culturing techniques. Such an approach could streamline compound screening and has the potential to expand the physiological and taxonomic diversity in our culture collections.

In addition to whole-cell-based detection, incorporation of azide-modified amino acids into proteins offers the possibility to physically enrich proteins that have been newly expressed during the time of incubation. By conjugating AHA-labelled proteins with alkyne-modified biotin reagents it is possible to separate those proteins on streptavidin- or neutravidin-coated affinity chromatography columns (Dieterich *et al*., 2006; 2007[Bibr b23],[Bibr b24]; Szychowski *et al.*, 2010). In theory, a single AHA residue is sufficient to separate the respective protein from the pre-existing protein fraction. This strategy has some advantages over protein stable isotope probing, which relies on the incorporation of comparatively high amounts of isotopic label for successful separation and identification of individual proteins (Jehmlich *et al.*, 2010; von Bergen *et al.*, 2013). Physical separation of the *de novo* synthesized fraction of the proteome before mass spectrometric sequencing has the potential to yield higher sequencing coverage of newly made proteins than may be observed in the bulk metaproteome.

Aside from its application to the study of protein expression, we expect click chemistry to have widespread utility in the microbial ecology field. For example, recent studies have demonstrated the successful incorporation of diverse bioorthogonal compounds into other major classes of biomolecules, including sugars (e.g. Saxon *et al.*, 2002; Banerjee and Carrico, 2011), lipids (e.g. Kho *et al.*, 2004; Neef and Schultz, 2009) and nucleic acids (e.g. Jao and Salic, 2008; Salic and Mitchison, 2008). We anticipate that the adaptation of these methods to complex, multi-species environmental samples will considerably expand the molecular toolbox for microbial ecologists studying uncultured and cultured microorganisms alike.

## Experimental procedures

### Chemicals

*L*-2-amino-4-azidobutanoic acid (AHA) was synthesized, purified and quality controlled according to established protocols (Link *et al.*, 2007) with the following slight modifications: ethyl acetate washing (step 11 in the original protocol) was performed twice to remove all CuSO_4_; after adding the Dowex resin (Sigma Aldrich) to the chromatography column (step 14) the resin was washed with 200 ml methanol and then 400 ml water before conditioning took place as described (step 15); AHA was eluted with 1 M NH_4_OH until the effluent was basic (∼ 100 ml; step 18); then, the column was rinsed with an additional 100 ml of 1 M NH_4_OH and the effluent recycled a few times to harvest all of the amino acid (step 18). In later stages of the project, commercially available AHA (Iris Biotech) was used. Tris[(1-hydroxypropyl-1*H*-1,2,3-triazol-4-yl)methyl]amine (THPTA), used as ligand in copper-catalyzed click reactions, was synthesized according to a published protocol (Hong *et al.*, 2009), but is also commercially available (e.g. Sigma). Currently, prices of AHA and THPTA offered by the above suppliers are lower than the costs associated with in-house synthesis.

### Culturing

Four pure and two enrichment cultures were selected to test the BONCAT method with a range of phylogenetically and metabolically diverse microorganisms. *Escherichia coli* K12 was grown at either 32 or 37°C (as indicated below) at 150 r.p.m. horizontal shaking in M9 minimal medium: 0.5 g NaCl, 2.0 g glucose, 1.0 g NH_4_Cl, 12.8 g Na_2_HPO_4_ × 7 H_2_O, 3.0 g KH_2_PO_4_, 492 mg MgSO_4_ × 7 H_2_O, 11 mg CaCl_2_ and 100 mg thiamine per 1 L of deionized water. Incubations in LB medium were performed at 37°C and 150 r.p.m.

*Paracoccus denitrificans* PD1222 was grown under denitrifying conditions at 32°C at 100 r.p.m. horizontal shaking in heterotrophic medium containing 0.5 g Na-citrate × H_2_O, 1.0 g (NH_4_)_2_SO_4_, 1.8 g KNO_3_, 7.0 g K_2_HPO_4_, 3.0 g KH_2_PO_4_, 100 mg MgSO_4_, 50 mg FeSO_4_ × 7 H_2_O, 100 mg thiamine and 1.28 ml ethanol per L. 1 ml trace element solution SL-10 (http://www.dsmz.de) and 1 ml vitamin solution (see medium 141, http://www.dsmz.de) were added. Media were degassed at 100°C for 20 min before the vials were sealed with butyl rubber stoppers, the headspace flushed with N_2_ and the vials autoclaved.

*Desulfovibrio alaskensis* (formerly *Desulfovibrio desulfuricans*) G20 was grown in medium 383 (prepared as recommended, http://www.dsmz.de) on 20 mM lactate at 37°C with 150 r.p.m. horizontal shaking.

*Methanosarcina acetivorans* C2A was grown at 33°C without agitation [tubes were shaken before optical density (OD) measurements, OD_600_] in an artificial seawater medium that contained per litre: 20.45 g NaCl, 136 mg KH_2_PO_4_, 147 mg CaCl_2_ × 2 H_2_O, 3.05 g MgCl_2_ × 6 H_2_O, 535 mg NH_4_Cl, 2.52 g NaHCO_3_, 360 mg Na_2_S × 9 H_2_O, 1 mg resazurin, as well as 1 ml of each SL-10 trace elements and medium 141 vitamin solution (http://www.dsmz.de). Cultures were supplemented with 0.5% (v/v) of both methanol and trimethylamine and incubated under 80% H_2_, 20% CO_2_ headspace at 207 kPa (30 psi) pressure.

Methanotrophic enrichments from marine sediment and propane-oxidizing enrichments from stream sediments were grown with a modified nitrate mineral salt medium at room temperature (RT) with mild horizontal shaking (20 rpm) in the dark (Tavormina *et al*., in review). The headspace contained 45% N_2_, 30% CH_4_ and 25% lab air or 50% propane, 50% lab air respectively.

### AHA incubations of cultures

AHA was added to log-phase cultures (assessed by optical density, OD_600nm_) using 100, 10 or 1 mM stock solutions yielding final concentrations of 1000, 100 or 10 μM respectively. Stock solutions were prepared in sterile filtered (0.2 μm) nanopure water and had a final pH of 7.0. AHA stock solutions for anaerobic incubations were sparged with N_2_ for 5 min. In addition, control incubations supplemented with water or *L*-Met (0.1 or 1 mM) were performed. The final volumes of all incubations for a given set of experiments were identical and ranged from 3 to 15 ml, depending on the cell density of the respective culture. All AHA and control incubations were run in duplicate. The optical densities (OD_600_) of cultures were regularly measured and growth rates compared. We did not observe an effect of AHA on the growth rate of any culture. After 0.2–1.0 generations (depending on growth rates and maximum cell densities of the individual cultures) incubations were stopped and cultures processed as described below.

To test on the effect of the protein synthesis inhibitor Camp on AHA incorporation in *E. coli* cells, we incubated an early log-phase *E. coli* K12 culture in the absence or presence of AHA (1 mM) and the absence or presence (290 mg l^−1^) of the antibiotic. After the control cells (i.e. *E. coli* grown with AHA but without Camp) had been grown for ∼ 0.8 generations all samples were chemically fixed (see below).

In the heat-shock experiment, AHA (1 mM final) was added to an early log-phase *E. coli* K12 culture and 3 ml aliquots were immediately transferred into preheated sterile glass vials. Vials were incubated for 30 min at 42°C without agitation. At the start of the experiment (after AHA addition, but before transfer to 42°C) as well as after 2, 4, 7, 12, 15 and 30 min, the entire volume of two replicate cultures was sampled. A single control without AHA was sampled after 30 min.

To compare AHA-labelling with ^15^NH_3_ uptake, a culture of *E. coli* K12 (grown overnight) was inoculated into M9 minimal medium and incubated for 3 h at 32°C with 150 r.p.m. horizontal shaking in the absence or presence of 1 mM AHA and 1.87 mM ^15^NH_4_Cl (Cambridge Isotope Labs; ^15^NH_4_^+^ constituted 9% of the total ^14+15^NH_4_^+^ pool).

At the end of each experiment, 1/3 of the culture volume was chemically fixed using 3% FA in 1× PBS for 60 min at RT, except for *M. acetivorans*, which was fixed in 3% FA in 1.5× PBS for 2.5 h at 4°C. After fixation, samples were centrifuged for 5 min at 16 100 *g* and the pellets washed three times with 1× PBS to remove free AHA. Pellets were resuspended in 50% EtOH in 1× PBS and stored at −20°C. The remaining 2/3 of the culture was centrifuged for 5 min at 5150 *g* (vol. > 4 ml) or 16 100 *g* (vol. < 4 ml), the supernatant (SN) discarded, and the cell pellet flash-frozen in liquid N_2_. Samples were stored at −80°C until further analysis.

### Physiological experiments with a methanotrophic enrichment culture

To visualize the dependency of the enriched *Methylococcaceae* sp. WF1 on methane, we incubated the methanotrophic enrichment culture for 2.5 days in the absence of methane (energy starvation). Then, 3 ml aliquots of the culture were incubated in duplicate either with (i) 1 mM AHA and 30% methane in the headspace, (ii) 1 mM AHA without methane or (iii) 30% methane in the headspace without AHA (this last incubation was not performed in replicate). 0.5 ml aliquots were removed after 4, 9 and 26 h. Cells were pelleted and cultures fixed for 1 h in 3% FA in 1× PBS at RT. Washing steps and cell storage were performed as described for the other cultures (see above).

To test whether AHA-labelled WF1 cells can be detected in a complex sample, aliquots of the culture that been incubated under the above described conditions were spiked into a FA-fixed sample of marine methane seep top-layer sediment (not the original sample from which WF1 had been enriched from) and the sample thoroughly mixed. To separate cells from the sediment matrix, samples were sonicated on ice for 30 s with a sonicating wand (sonifier 150; Branson) at 6 W. One volume of ice-cold percoll (GE Healthcare Life Sciences) was added to the bottom of the tube and the samples centrifuged for 20 min at 16 100 *g* at 4°C. Afterwards, the liquid SN atop the percoll was transferred into a new tube, 1 volume 1× PBS added, and the sample centrifuged for 5 min at 16 100 *g* at RT. The pelleted cells were resuspended in 50% EtOH in 1× PBS and stored at −20°C.

### AHA incubations of environmental samples

Three complex samples (3–15 ml each) were incubated in autoclaved glass tubes in the absence or presence (1 mM) of AHA: (i) saliva and biofilm scraped from the tongue of one of the authors; (ii) freshwater and (iii) top-layer (upper 1 cm) sediment from the ‘Lily pond’ on the Caltech campus. Oral samples were incubated for 6 h at 32°C with 100 r.p.m. horizontal shaking. We do not expect that these conditions are representative of the conditions microbes face within their human host. Rather, this experiment is intended to serve as a proof of principle demonstration and open the way for future experiments on human-associated microbes. Sediment samples (pH 7.0; 11°C) were transferred into test tubes, which were then closed with rubber stoppers and incubated *in situ* for 7.5 h half-submerged in water. Freshwater samples (pH 6.7; 21°C), containing aggregations of purple sulfur bacteria visible with the naked eye, were transferred into test tubes, closed with septa and incubated in the presence of AHA for 4 h. Incubations were performed during daytime, i.e. under natural light condition, and in duplicate. Aliquots of samples were fixed in 3% FA in 1× PBS for 1 h at RT. Sulfide (i.e. H_2_S plus HS^−^) concentrations in water and sediment samples were determined using the cline assay (Cline, 1969), which had a detection limit of 60 μM.

### Alkyne-conjugated dyes

Several alkyne-modified dyes were tested for their suitability for protein click labelling. Dyes were purchased from the following companies: Invitrogen (Oregon Green 488 alkyne), Lumiprobe (Cy3 alkyne) and ClickChemistryTools (Carboxyrhodamine 110 Alkyne, DBCO-PEG_4_-Carboxyrhodamine 110, DBCO-PEG4-Tetramethylrhodamine and Eosin-alkyne).

### Cu(I)-catalyzed click labelling of chemically fixed microbial cells

The azide-alkyne [3 + 2] cycloaddition (click) reaction requires a copper-catalyst, which is typically prepared with a chelating ligand, e.g. tris[(1-hydroxypropyl-1H-1,2,3-triazol-4-yl)methyl]amine (THPTA; Hong *et al.*, 2009) or similar compounds (Besanceney-Webler *et al.*, 2011). These molecules help to keep the metal in its Cu(I) oxidation state. Because of the instability of Cu(I) under standard lab air conditions, CuSO_4_ is usually added in large excess (∼ 100 μM) and in the presence of the reducing agent sodium ascorbate (∼ 5 mM). Furthermore, aminoguanidine (∼ 5 mM) is added to the reaction to inhibit protein cross-linking and precipitation (Hong *et al.*, 2009 and references therein). Dye was used at 25 μM final concentration.

The protocol involved the following steps: fixed samples were immobilized on glass slides; dried in a hybridization oven (46°C); dehydrated and permeabilized by placing slides for 3 min each in 50, 80 and 96% ethanol; and then dried using pressurized air. Then, 1.25 μl of a 20 mM CuSO_4_ (in water; stored at 4°C) solution, 2.50 μl of 50 mM THPTA (in water; stored at 4°C) and 0.30 μl of alkyne dye [in dimethyl sulfoxide (DMSO) or a 1:1 DMSO : water mix] were mixed and allowed to react for 3 min at RT in the dark (i.e. dye premix). In the meantime, 12.5 μl of freshly prepared 100 mM sodium ascorbate (Sigma-Aldrich; freshly made in water; 5 mM final) and 12.5 μl of 100 mM aminoguanidine hydrochloride (Sigma-Aldrich; freshly made in water; 5 mM final) were added to 221 μl 1× PBS (pH 7.4). Then, the dye premix was added to this solution, the tube inverted once (do not mix by vortex to maintain reducing conditions), and samples were covered by 30 μl of solution. Slides were transferred into a humid chamber (water on tissue paper) and incubated in the dark at RT for 30 min. Afterwards, slides were washed three times for 3 min each in 1× PBS, treated with an increasing ethanol series (3 min each in 50, 80 and 96% ethanol) and air-dried.

In addition to on-slide-labelling, on-filter and in-solution click labelling were attempted. For on-filter click labelling, samples of pure cultures were immobilized on 0.2 μm GTBP filters (Millipore). All subsequent steps were identical to the protocol described above.

For click labelling in solution, samples were resuspended in 221 μl 1× PBS, to which solutions were added as described above. Tubes were inverted once and then incubated in the dark at RT for 30 min. Afterwards, samples were washed three times with 1x PBS, and then three times in an increasing ethanol series (50, 80 and 96%). Each washing step was followed by pelleting samples via centrifugation for 5 min at 16 100 *g* at RT. Finally, cells were resuspended in 50% ethanol in 1× PBS, transferred onto a glass slide, dried at 46°C, DAPI/Citifluor (Science Services) mounted, and microscopically analyzed.

### Strain-promoted click labelling of chemically fixed microbes

Strain-promoted click chemistry is different from the above approach as it does not depend on the presence of a catalyst (Beatty *et al.*, 2010; Chang *et al.*, 2010). Instead, the reaction rate is increased by using strained dibenzocyclooctyne (DBCO) molecules to which fluorescence dyes have been conjugated via a polyethylene glycol linker (Supporting Information Fig. S1EF).

Samples were immobilized on glass slides, dried in a hybridization oven (46°C), dehydrated and permeabilized by placing slides for 3 min each in 50, 80 and 96% ethanol, and then dried using pressurized air. Slides were incubated for 1 h in 100 mM 2-chloroacetamide in Tris/HCl (pH 7.4) at 46°C in the dark to block free thiols. Then, dyes were directly added to this solution to reach final concentrations of 100 nM to 1 μM (using a 5 mM stock solution). Click reactions were carried out for 30 min at 46°C in the dark. To remove unbound dye, different washing protocols were tested, of which the following proved generally successful: after click labelling, slides were washed for 10 min at 48°C in 1× PBS (pH 7.4) and then transferred to a solution of 50% DMSO in 1x PBS (RT). Slides were incubated for 20 min and then washed three times for 3 min each in 1× PBS. Last, slides were washed/dehydrated in an increasing ethanol series (3 min each in 50, 80 and 96% ethanol) and air-dried. Slides were mounted with DAPI/Citifluor and microscopically analyzed, or, alternatively, FISH was performed. If incubation at 46/48°C is logistically not possible, reactions and washing can also be performed at RT or 37°C. However, this will result in lower signal-to-noise ratios.

### FISH

To establish the BONCAT-FISH protocol, artificial mixes of pure and enrichment cultures were immobilized on glass slides. Due to the low salt concentration of the buffers used for click chemistry, FISH was always performed after click chemistry-mediated labelling of AHA-containing proteins to maintain specificity during oligonucleotide probe hybridization. This order minimizes the potential for dissociation of the hybridized probes from their target rRNAs and possible re-association with non-target rRNAs in the low stringency buffer used in click chemistry. After BONCAT had been performed and slides had been dehydrated via an increasing ethanol series (3 min in each 50, 80 and 96% ethanol), 16S and 23S rRNA-targeted FISH was carried out following established protocols. Briefly, samples were hybridized with Cy3- and Cy5-labelled oligonucleotide probes for 1.5–4 h (depending on the sample) in a humid chamber at 46°C. Formamide concentrations in the buffer were as recommended: 10–60% for probe mix EUB338 I-III (Amann *et al.*, 1990; Daims *et al.*, 1999) and control probe NonEUB338 (Wallner *et al.*, 1993), 20% for probe Alf968 (Neef *et al.*, 1999) and 35% for probes Bet42a, Gam42a (both used with unlabelled competitors probes; Manz *et al.*, 1992) and Arch915 (Stahl and Amann, 1991). For probe MetI-444, used to detect *Methylococcaceae* sp. WF1 in one of our enrichment cultures, a formamide concentration of 35% was used, while 60% was originally recommended (Losekann *et al.*, 2007). However, the sediment sample in which this probe had been recently applied (Losekann *et al.*, 2007) is substantially more complex than our enrichment culture. Besides our target gamma proteobacterium, at the time of analysis the sample hosted up to five other bacterial species, each at abundances < 1% of the total population (based on DAPI counts). We never observed more than one FISH-positive morphotype (coccoid methanotroph WF1 cells, Fig. 4F), consistent with the *in silico* prediction of several mismatches to non-target rRNAs. When applied to the prestine sediment sample used in our spiking experiment, we did not observe MetI-444 (35% formamide)-positive cells. After hybridization, slides were washed for 10 min in pre-warmed washing buffer at 48°C before they were dipped into 4°C deionized water to remove salts. After slides were dried with pressurized air, they were mounted with DAPI/Citifluor and analyzed via epifluorescence microscopy. Fluorescence images were analyzed using imageJ (NIH).

### nanoSIMS analysis of BONCAT-labelled cells

An artificial mix of microbes, which consisted of AHA- and ^15^N-labelled *E. coli*, the AHA-labelled methanotrophic enrichment, as well as unlabelled aliquots of *P. denitrificans*, *D. variabilis* and *Methanosarcina vanielli* (provided by Hiroyuki Imachi), was immobilized on silicon wafers. Cu(I)-catalyzed click labelling was performed as described above using the fluorine-containing dye Oregon Green 488 alkyne (Supporting Information Fig. S1A), followed by FISH using probe MetI-444 (see above). Biomass was microscopically imaged and analyzed via the nanoSIMS 50 L (Cameca) at Caltech's Microanalysis Center. Areas were pre-sputtered using high Cs^+^ beam currents before measurements were performed. Images were acquired with a 3 pA Cs^+^ ion primary beam, focused to a spot size of 100 nm. Analyzed regions were 30 × 30 or 40 × 40 μm in area and were rastered using a resolution of 512 × 512 pixels and a dwell time of 6.8 ms. The nanoSIMS was run in multi-collector mode with a mass resolving power of ∼ 7000 with electron multipliers positioned to detect ^19^F^-^, ^12^C^-^, ^12^C^14^N^-^, ^12^C^15^N^-^, and ^31^P^-^. Data was analyzed using the limage software (Cameca). Definitions of ROI were guided by the ^12^C^15^N^-^ signal of *E. coli*, the FISH signal of methanotroph WF1 and the ^12^C^-^ signal, which was used as an indicator of biomass location. Due to the physical proximity of cells to each other, it was not always possible to define ROI for individual cells. Thus, the number of cells per ROI varies between 1 and 5. Similar experiments were attempted using a Br-containing dye (Eosin) for nanoSIMS analyses. However, this dye was found to be incompatible with BONCAT due to the difficulty of removing unbound dye from the cell (Supporting Information Fig. S1D).

### Extraction, labelling and purification of proteins

Frozen pellets were resuspended in 1 ml extraction buffer [1% sodium dodecyl sulfate (SDS), 50 mM Tris, 150 mM NaCl, 100 mM EDTA, 1 mM MgCl_2_ at pH 8.4] and boiled in a water bath for 30 min. Note that at this point, no reducing agent, such as dithiotreitol (DTT) or β-mercaptoethanol, was added because they would reduce the azide group of AHA. After cell lysis, samples were allowed to cool for 5 min at RT before they were centrifuged at 16 100 *g* for 5 min. The SN was transferred into a new tube and proteins were quantified using the bicinchoninic acid (BCA) protein assay according to the manufacturer's protocol (Thermo Scientific). If not immediately processed, crude protein extracts were stored at −20°C. To label AHA-containing proteins, a volume equivalent to 100–250 μg of protein was transferred into a new tube and 2-chloroacetamide was added (100 mM final concentration). Tubes were shaken for 1 h at RT in the dark before dye DBCO-PEG_4_-Carboxyrhodamine 110 (Supporting Information Fig. S1E) was added to a final concentration of 10 μM. Tubes were shaken for 30 min at RT in the dark before 1 mM AHA was added to stop the reaction. Proteins were extracted using a 600:150:400 μl methanol : chloroform : water mix and the pellet washed three times with 1 ml of pure methanol. All centrifugation steps were done at 16 100 *g* at RT. After the final wash, a volume of ∼ 200 μl was left in the tube and the sample was desiccated using a vacuum centrifuge. Extracted proteins were processed immediately or stored at 4°C for up to 3 days.

### Protein gels

Pellets were resuspended in loading buffer to which 200 mM DTT had been freshly added. Proteins were denatured at 65°C for 5 min before 5–10 μg of protein were loaded and run at a constant voltage of 175 V for 45 min on NuPAGE Novex 4–12% Bis-Tris gels (Invitrogen) using MES running buffer (50 mM of 2-(*N*-morpholino)ethanesulfonic acid, 50 mM of Tris Base, 0.1% of SDS, 1 mM of EDTA, pH 7.3; chemicals from Sigma-Aldrich). PageRuler protein ladder (Thermo Scientific) was used as a marker of molecular weight. After electrophoresis, gels were fixed for 20 min in a 1:4:5 acetate : methanol : water mix before being washed three times in deionized water for 5 min each under horizontal shaking. Gels were scanned using a Typhoon laser scanner (GE Healthcare Life Sciences) at an excitation wavelength of 532 nm to visualize fluorescently labelled proteins. Afterwards, protein gels were stained for 1 h using GelCode Blue Stain Reagent (Thermo Scientific), washed for 1 h in deionized water under horizontal shaking, and imaged.

### Extraction of labelled gel bands and protein sequencing

BONCAT-labelled proteins from a methanotrophic enrichment as well as from an *E. coli* culture were run on a protein gel as described above. The gel was stained for 1 h in colloidal Coomassie stain (Invitrogen) at RT and fluorescently labelled bands were excised with a scalpel, and each excised band was finely diced and transferred to an Eppendorf tube. To each gel piece 100 μl of ammonium bicarbonate (AB; filtered through a 0.2 μm filter after preparation) was added and incubated for 5 min (all steps were done at RT). Buffer was removed with a pipette and 50 μl of a 1:1(v/v) 50 mM AB/acetonitrile (ACN) was added to the gel pieces and incubated for 5 min. These de-staining steps were repeated twice to remove all Coomassie stain. Then, 25 μl of 50 mM AB and 50 μl of freshly prepared 10 mM DTT in 100 mM AB were added to the gel pieces and incubated at 50°C for 30 min. Afterwards, the solution was removed and 25 μl of 50 mM AB was added to the gel. To this mixture, 50 μl of freshly prepared 55 mM iodoacetamide in 100 mM AB was added and the gel pieces incubated in the dark for 20 min. Then, solution was removed, 100 μl of 50 mM AB was added and gel pieces incubated for 5 min. After removal of this solution, gel pieces were washed with 100 μl of ACN for 5 min. Then, 75 μl of 50 mM AB and 25 μl 6 ng μl^−1^ trypsin (prepared from a 100 ng μl^−1^ stock solution in 1 mM HCl) were added, and tubes incubated overnight at 37°C. Then, the SN was transferred into a new tube, and 100 μl of 1% formic acid, 2% ACN was added to the gel pieces. After incubation for 5 min, the solution was transferred to a vial containing SN. Gel pieces were washed with 100 μl of 1:1 (v/v) ACN : water, and after 5 min incubation the SN was again transferred. In a last washing step, 100 μl of 1% formic acid in ACN was added to the gel pieces, left for 5 min, and the SN transferred. The extracted peptides were dried using a vacuum centrifuge at 37°C, and afterwards dissolved in 0.2% formic acid. The dried peptides were resuspended in 80% ACN and separated from remaining gel pieces by purification via a C18 column. Peptides were sequenced using a liquid chromatography mass spectrometry system and raw data analyzed with the maxquant quantitative proteomics software (Cox and Mann, 2008) as recently described (Kalli *et al.*, 2013).

### Abiotic incubations of AHA

The following buffers were prepared anaerobically: 50 mM potassium phosphate pH 7.0, 100 mM potassium phosphate pH 8.0 and 100 mM carbonate pH 9.0. All buffers were prepared by mixing the appropriate amounts of KH_2_PO_4_/K_2_HPO_4_ and NaHCO_3_/Na_2_CO_3_ respectively. Ten volume per cent of D_2_O was added to all buffers for locking the NMR measurements. Each of the corresponding solutions (700 μl total) was mixed in an anaerobic chamber from a stock solution of 100 mM AHA (pH 7.0) and from a 100 mM Na_2_S stock solutions (adjusted to pH 7, 8 and 9 respectively). Each solution was transferred into a 5 mm NMR tube, closed with a septum and a NMR spectrum was recorded. After analysis, the NMR tubes were stored at RT in the anaerobic chamber until further measurements. For experiments at pH 9, samples were neutralized by adding 40 μl 1.0 M KH_2_PO_4_ directly into the NMR tube (pH 7.5) before measurement of the 77 h time point for easier comparison with the other spectra. An additional sample was prepared aerobically with 10 mM Na_2_S at pH 8.0. To test on the effect of high temperature, 10 mM AHA solutions in sterile water were adjusted to pH 5.0 and 7.1 and solutions transferred into 5 mm NMR tubes. Ten volume per cent of D_2_O were added, tubes closed with a septum and NMR spectra were recorded. After analysis, the tubes were incubated in a drying oven at 80 ± 3°C for 19.5 h and then again analyzed via NMR.

### NMR spectroscopy

^1^H-NMR spectra were recorded on a Varian 400 MHz spectrometer with a broadband auto-tune OneProbe (Varian). The spectra were recorded at 25°C without spinning (90° excitation pulse, 2.6 s acquisition time) and continuous wave pre-saturation of the water signal for 2 s was employed (4.6 s repetition rate). Sixty-four scans were recorded each using identical parameters for all samples. Spectra were processed with inmr 4.1.7 software (Nucleomatica) using manual phase correction, 0.1 Hz of line broadening and automatic baseline correction. The ratio of AHA to *L*-2,4-diaminobutyric acid was quantified via integration of the signal RC*H_2_*N_3_ at 3.4 p.p.m. (AHA) and RC*H_2_*NH_2_ at 2.95 p.p.m. (2,4-diaminobutyric acid). For incubations in the presence of sulfide, no decomposition products other than 2,4-diaminobutyric acid could be detected. The identity of 2,4-diaminobutyric acid was verified by reducing an aliquot of AHA with tris(2-carboxyethyl)phosphine (Staudinger reduction) and comparing its NMR spectrum in pH 8.0 buffer with the sulfide incubation spectra.

## Supporting Information

Fig S1 Comparative BONCAT of newly synthesized proteins in *E. coli* and structures of dyes used for (A-D) Cu(I)-catalyzed and (E-F) strain-promoted click chemistry. Exposure times for cells incubated in the presence or absence of AHA are identical for each dye and were chosen to yield approximately comparable signal intensities for AHA-containing cells. Gain and offset settings of the detector were kept constant for all conditions. Note that different dyes give highly divergent signal to noise ratios when washing conditions are identical. Because of these background problems the Eosin-dye, initially believed to be a potential alternative for detection of AHA incorporation via nanoSIMS, could not be used in any of our experiments. The scale bar applies to all photos and equals 10 μm. Chemical structures were obtained from the respective company websites (see main text). Abbreviations: DBCO, Dibenzocyclooctyne; PEG, Polyethylene glycol.

Fig S2 Visualization of newly made proteins in living (liveBONCAT; panels A & B) and chemically fixed (C, D) AHA-labelled *E. coli*. Exposure times were identical for all images. Note that *E. coli* cells in panels (A) and (B) exhibit atypical cell morphology and that substantially lower numbers of cells are fluorescently labelled despite cells from the same culture are analyzed. The scale bar applies to all photos and equals 10 μm.

Fig S3 Distributions and average contents of methionine in candidate proteins encoded in the genomes of five microbes analyzed via BONCAT. Note that the genome of *Methylococcaceae* sp. WF1 has not been closed yet.

Fig S4 BONCAT-based visualization of newly synthesized proteins combined with 16S rRNA-targeted FISH in an artificial mix of microbial cultures. A methanotrophic enrichment culture that had been incubated in the presence of AHA was mixed with normally grown cultures of *E. coli*, *P. denitrificans*, *Methanosarcina* sp., and a propane-oxidizing enrichment culture. Consecutively, BONCAT was performed using Oregon Green 488 alkyne (green), FISH was performed using Cy3 (red) and Cy5 (turquoise) labeled oligonucleotide probes, and biomass was stained using DAPI (blue). (A) Localization of probe MetI-444 (red), specific for a group of gamma proteobacterial methanotrophs, reveals that coccoid WF1 cells from the methanotrophic enrichment culture incorporated AHA into their proteins. WF1 cells are not detected by probe Gam42a (turquoise; used with unlabeled competitor Bet42a), which is specific for many but not all gamma proteobacteria, due to a discriminating mismatch in their 23S rRNA. (B) FISH controls taken at identical exposure times for the respective channels (Cy3-labelled NonEUB338; auto-fluorescence in Cy5 channel). For BONCAT controls of the individual cultures see Fig. 2. For probe details refer to main text. The scale bars equal 10 μm.

Fig S5 Complimentary images to the comparative BONCAT analyses of a methanotrophic enrichment culture using different detection settings. Please note that for samples incubated for > 9 h in the presence of AHA, fluorescent signal of WF1 cells saturated the sensor (see Fig. 6). These settings were chosen in order to visualize the difference in fluorescence intensity after 4 h of incubation between WF1 cells grown in the absence and presence of methane. For images shown in this panel, exposition settings were chosen based on the much stronger signal intensities observed for later samples. All photos in this figure were taken at identical microscopic settings. Scale bar equals 10 μm and applies to all images.

Fig S6 BONCAT and FISH controls for microbiome and environmental samples. (A) Results for samples incubated in the absence of AHA (no BONCAT signal). (B) Tests of the specificity of the FISH protocol using a Cy3-labelled NonEUB338 probe (no FISH signal). Camera settings to record BONCAT and FISH signals were identical to those used to image samples shown in Fig. 5B–D respectively. All scale bars equal 10 μm.

Fig S7 Reduction of AHA in the presence of high concentrations of sulfide and high pH. (A) Results for abiotic reactions of 1 mM AHA in the absence or presence (H_2_S plus HS^-^ equals 1, 2, or 10 mM) of sulfide at pH 8.0 (HPO_4_^-^ buffered) and pH 9.0 (HCO_3_^-^ buffered). At pH 8 and 9, AHA is slowly reduced with a reduction rate proportional to the sulfide concentration. At pH 7, the pH of the sediment sample studied here (sulfide concentration ≤ 60 μM), sulfide has no detectable effect on AHA for up to at least 8 days (data not shown). The stability of AHA itself is not influenced by pH, and sulfide has no effect on AHA at pH ≤ 7. AHA and its reduction product with sulfide, *L*-2,4-diaminobutyric acid (B), were analyzed via NMR spectroscopy.

Fig S8 Visualization of clusters of protein-synthesizing gammaproteobacterial freshwater bacteria (identified via FISH-probe Gam42a (red), which was used with its competitor). The scale bar equals 20 μm.

Table S1 (A) Proteins identified via MS sequencing of gel-extracted proteins from methanotrophic enrichment culture WF1, ranked by abundance. (B) Proteins identified via MS sequencing of gel-extracted proteins from a heat-stressed E. coli K12 culture, ranked by abundance.

File S1 liveBONCAT: Attempting to visualize protein synthesis in living cells. Strain-promoted click labeling of living microbes.
