# Supplementary figures and images for: In situ visualization of newly synthesized proteins in environmental microbes using amino acid tagging and click chemistry
Source: Environ Microbiol. 2014 Apr 1;16(8):2568–90. doi: 10.1111/1462-2920.12436 (PMC4122687; doi:10.1111/1462-2920.12436)

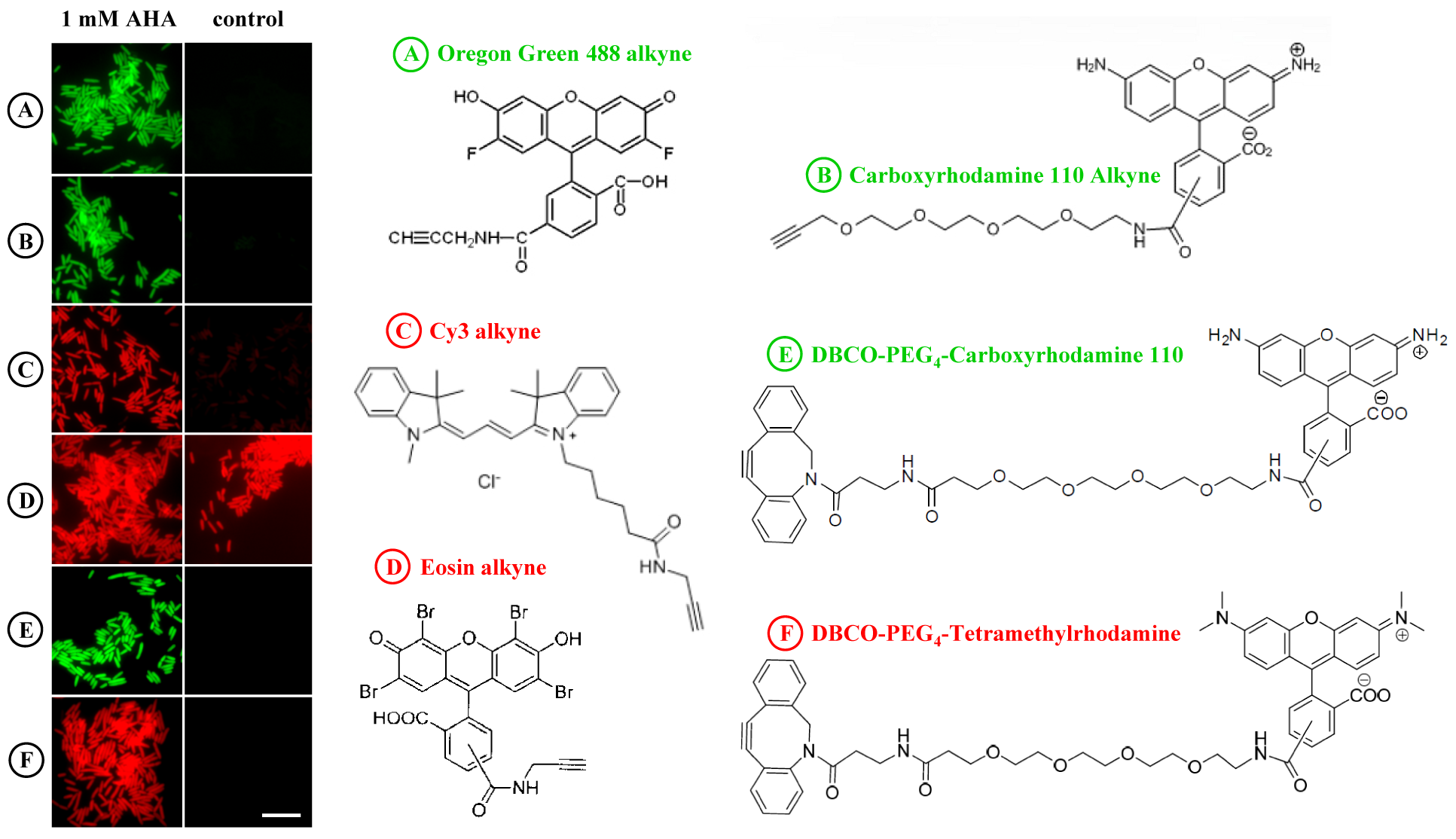

Supplement: Fig S1 — Comparative BONCAT of newly synthesized proteins in E. coli and structures of dyes used for (A-D) Cu(I)-catalyzed and (E-F) strain-promoted click chemistry. Exposure times for cells incubated in the presence or absence of AHA are identical for each dye and were chosen to yield approximately comparable signal intensities for AHA-containing cells. Gain and offset settings of the detector were kept constant for all conditions. Note that different dyes give highly divergent signal to noise ratios when washing conditions are identical. Because of these background problems the Eosin-dye, initially believed to be a potential alternative for detection of AHA incorporation via nanoSIMS, could not be used in any of our experiments. The scale bar applies to all photos and equals 10 μm. Chemical structures were obtained from the respective company websites (see main text). Abbreviations: DBCO, Dibenzocyclooctyne; PEG, Polyethylene glycol. [file emi0016-2568-SD1.tif]

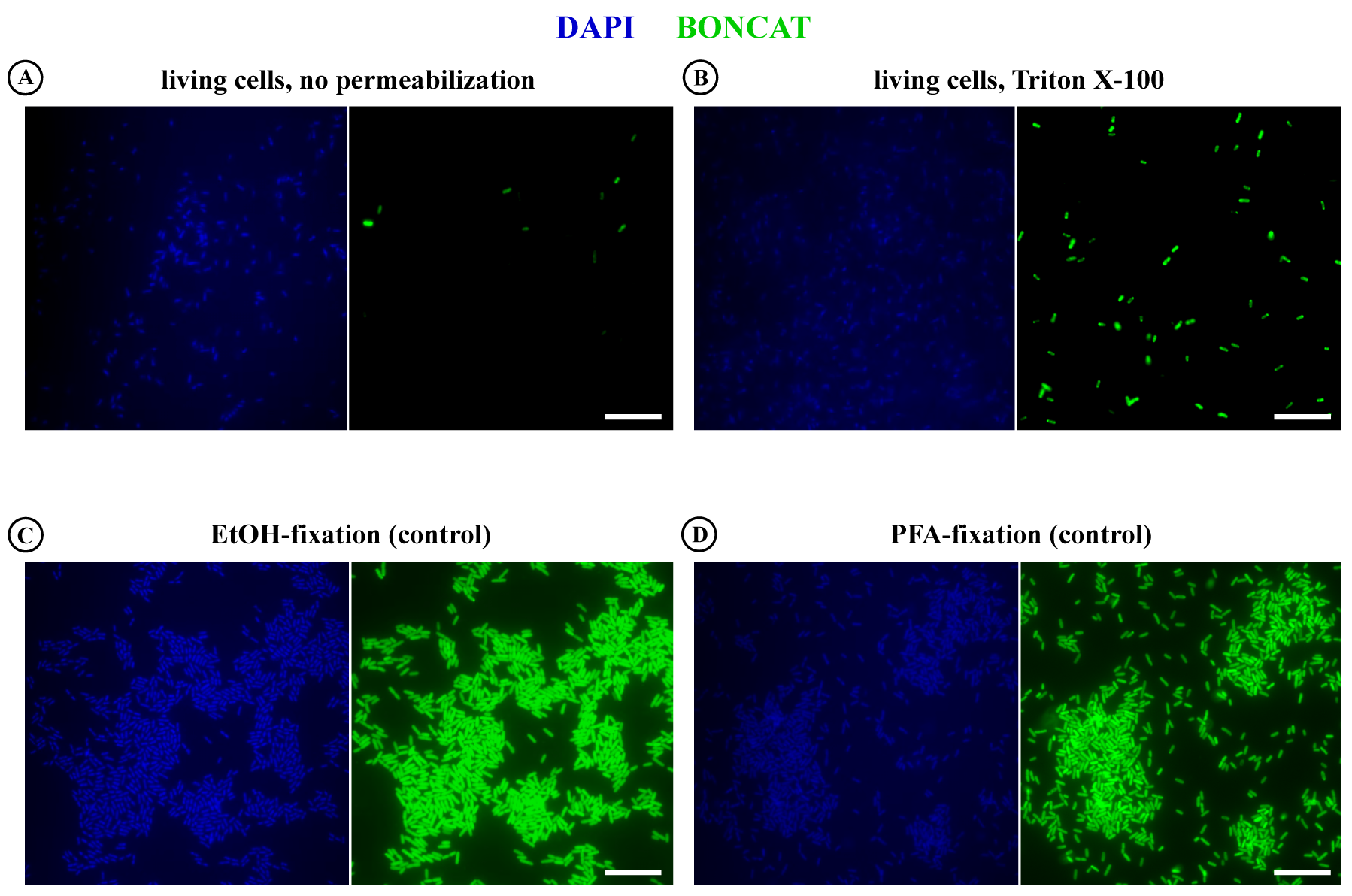

Supplement: Fig S2 — Visualization of newly made proteins in living (liveBONCAT; panels A & B) and chemically fixed (C, D) AHA-labelled E. coli. Exposure times were identical for all images. Note that E. coli cells in panels (A) and (B) exhibit atypical cell morphology and that substantially lower numbers of cells are fluorescently labelled despite cells from the same culture are analyzed. The scale bar applies to all photos and equals 10 μm. [file emi0016-2568-SD2.tif]

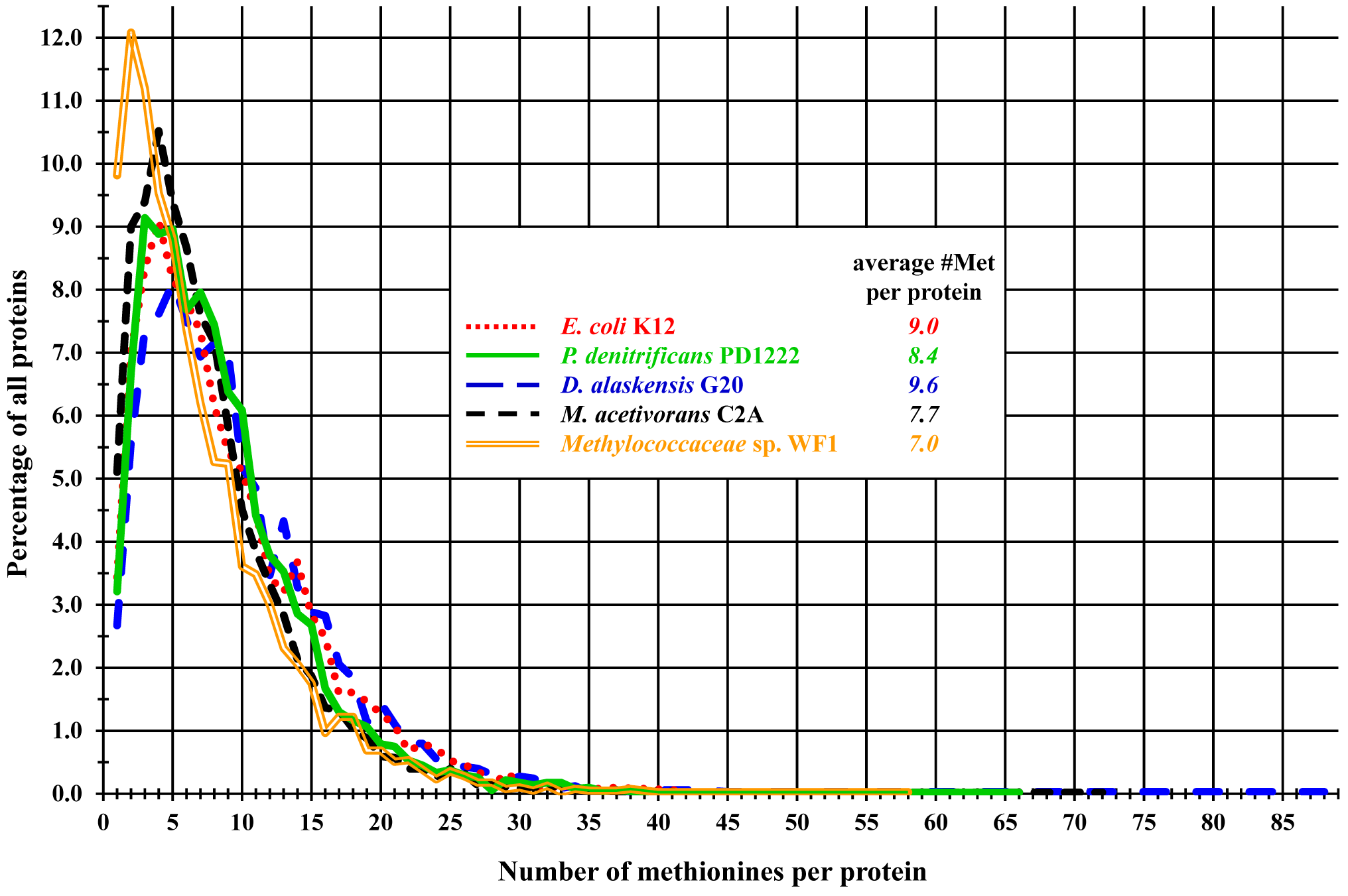

Supplement: Fig S3 — Distributions and average contents of methionine in candidate proteins encoded in the genomes of five microbes analyzed via BONCAT. Note that the genome of Methylococcaceae sp. WF1 has not been closed yet. [file emi0016-2568-SD3.tif]

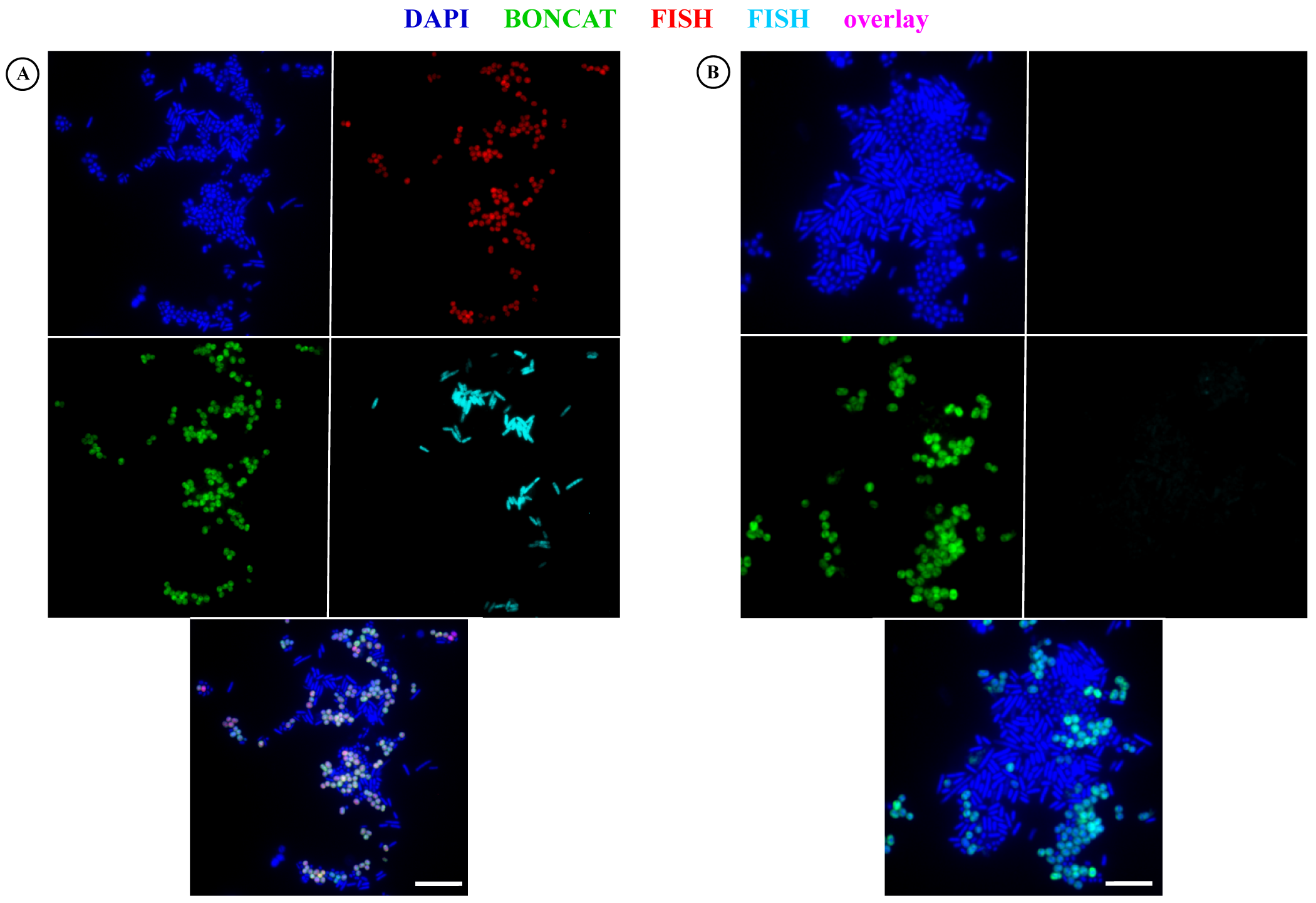

Supplement: Fig S4 — BONCAT-based visualization of newly synthesized proteins combined with 16S rRNA-targeted FISH in an artificial mix of microbial cultures. A methanotrophic enrichment culture that had been incubated in the presence of AHA was mixed with normally grown cultures of E. coli, P. denitrificans, Methanosarcina sp., and a propane-oxidizing enrichment culture. Consecutively, BONCAT was performed using Oregon Green 488 alkyne (green), FISH was performed using Cy3 (red) and Cy5 (turquoise) labeled oligonucleotide probes, and biomass was stained using DAPI (blue). (A) Localization of probe MetI-444 (red), specific for a group of gamma proteobacterial methanotrophs, reveals that coccoid WF1 cells from the methanotrophic enrichment culture incorporated AHA into their proteins. WF1 cells are not detected by probe Gam42a (turquoise; used with unlabeled competitor Bet42a), which is specific for many but not all gamma proteobacteria, due to a discriminating mismatch in their 23S rRNA. (B) FISH controls taken at identical exposure times for the respective channels (Cy3-labelled NonEUB338; auto-fluorescence in Cy5 channel). For BONCAT controls of the individual cultures see Fig. 2. For probe details refer to main text. The scale bars equal 10 μm. [file emi0016-2568-SD4.tif]

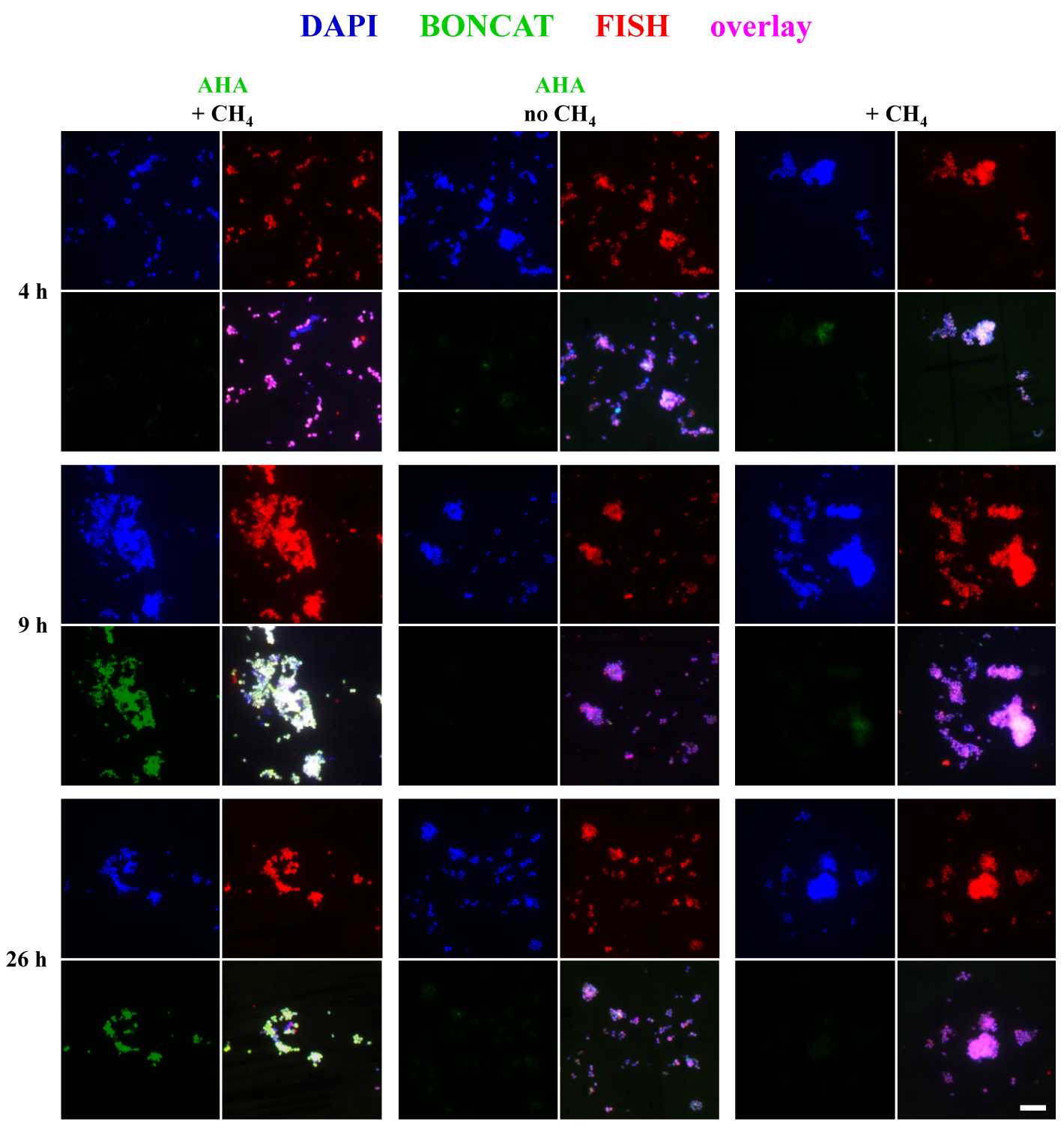

Supplement: Fig S5 — Complimentary images to the comparative BONCAT analyses of a methanotrophic enrichment culture using different detection settings. Please note that for samples incubated for > 9 h in the presence of AHA, fluorescent signal of WF1 cells saturated the sensor (see Fig. 6). These settings were chosen in order to visualize the difference in fluorescence intensity after 4 h of incubation between WF1 cells grown in the absence and presence of methane. For images shown in this panel, exposition settings were chosen based on the much stronger signal intensities observed for later samples. All photos in this figure were taken at identical microscopic settings. Scale bar equals 10 μm and applies to all images. [file emi0016-2568-SD5.tif]

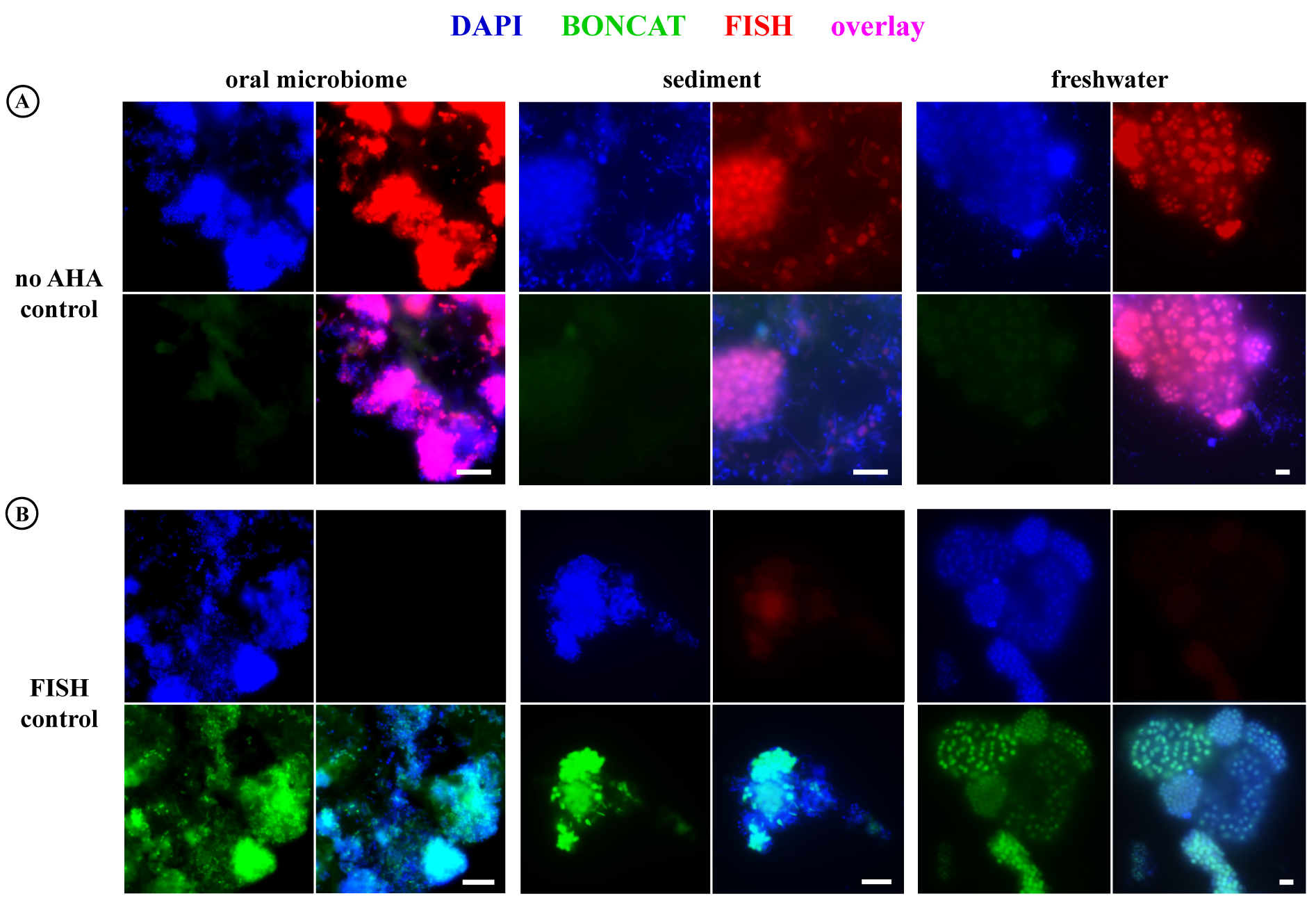

Supplement: Fig S6 — BONCAT and FISH controls for microbiome and environmental samples. (A) Results for samples incubated in the absence of AHA (no BONCAT signal). (B) Tests of the specificity of the FISH protocol using a Cy3-labelled NonEUB338 probe (no FISH signal). Camera settings to record BONCAT and FISH signals were identical to those used to image samples shown in Fig. 5B–D respectively. All scale bars equal 10 μm. [file emi0016-2568-SD6.tif]

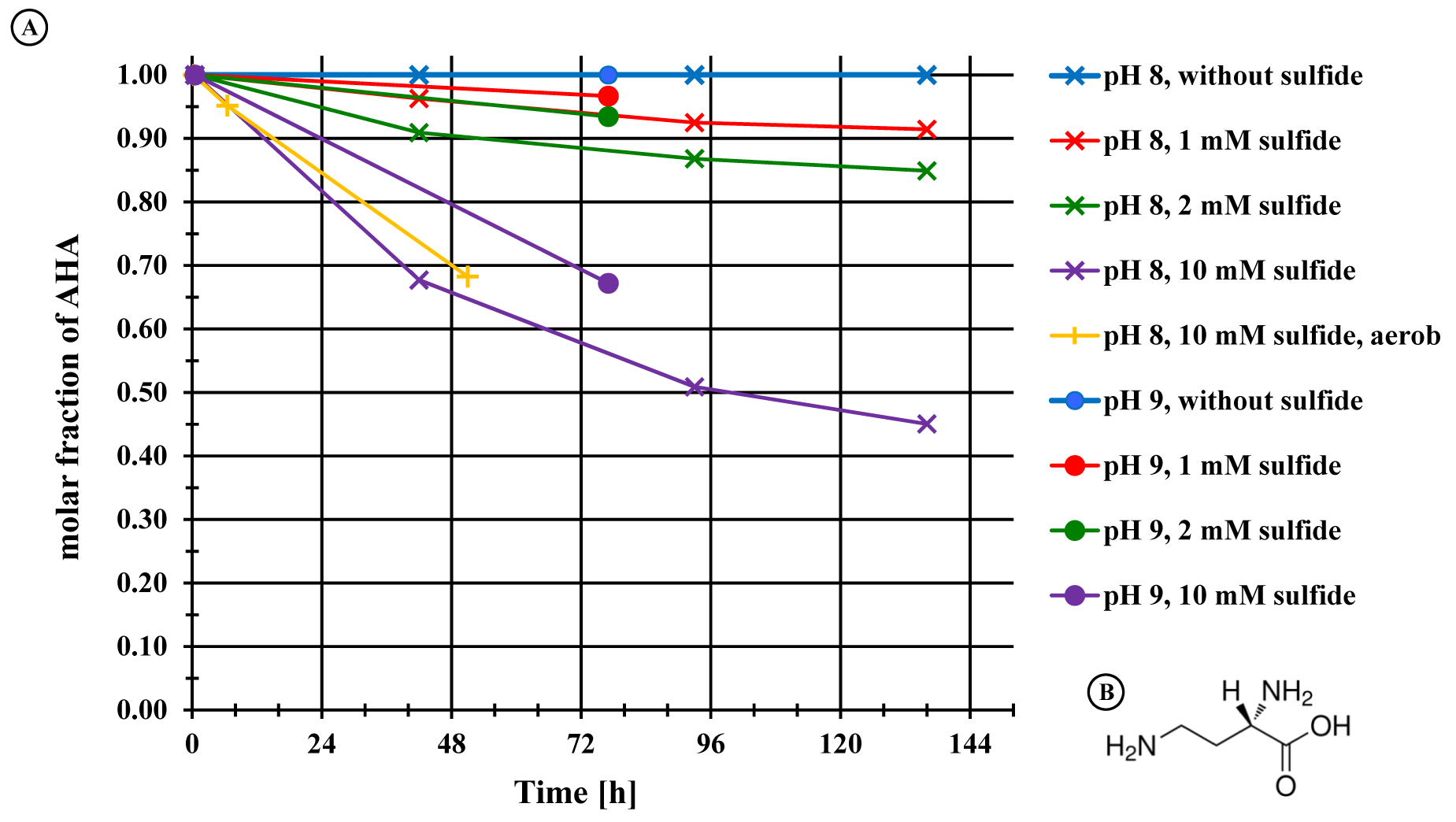

Supplement: Fig S7 — Reduction of AHA in the presence of high concentrations of sulfide and high pH. (A) Results for abiotic reactions of 1 mM AHA in the absence or presence (H2S plus HS- equals 1, 2, or 10 mM) of sulfide at pH 8.0 (HPO4- buffered) and pH 9.0 (HCO3- buffered). At pH 8 and 9, AHA is slowly reduced with a reduction rate proportional to the sulfide concentration. At pH 7, the pH of the sediment sample studied here (sulfide concentration ≤ 60 μM), sulfide has no detectable effect on AHA for up to at least 8 days (data not shown). The stability of AHA itself is not influenced by pH, and sulfide has no effect on AHA at pH ≤ 7. AHA and its reduction product with sulfide, L-2,4-diaminobutyric acid (B), were analyzed via NMR spectroscopy. [file emi0016-2568-SD7.tif]

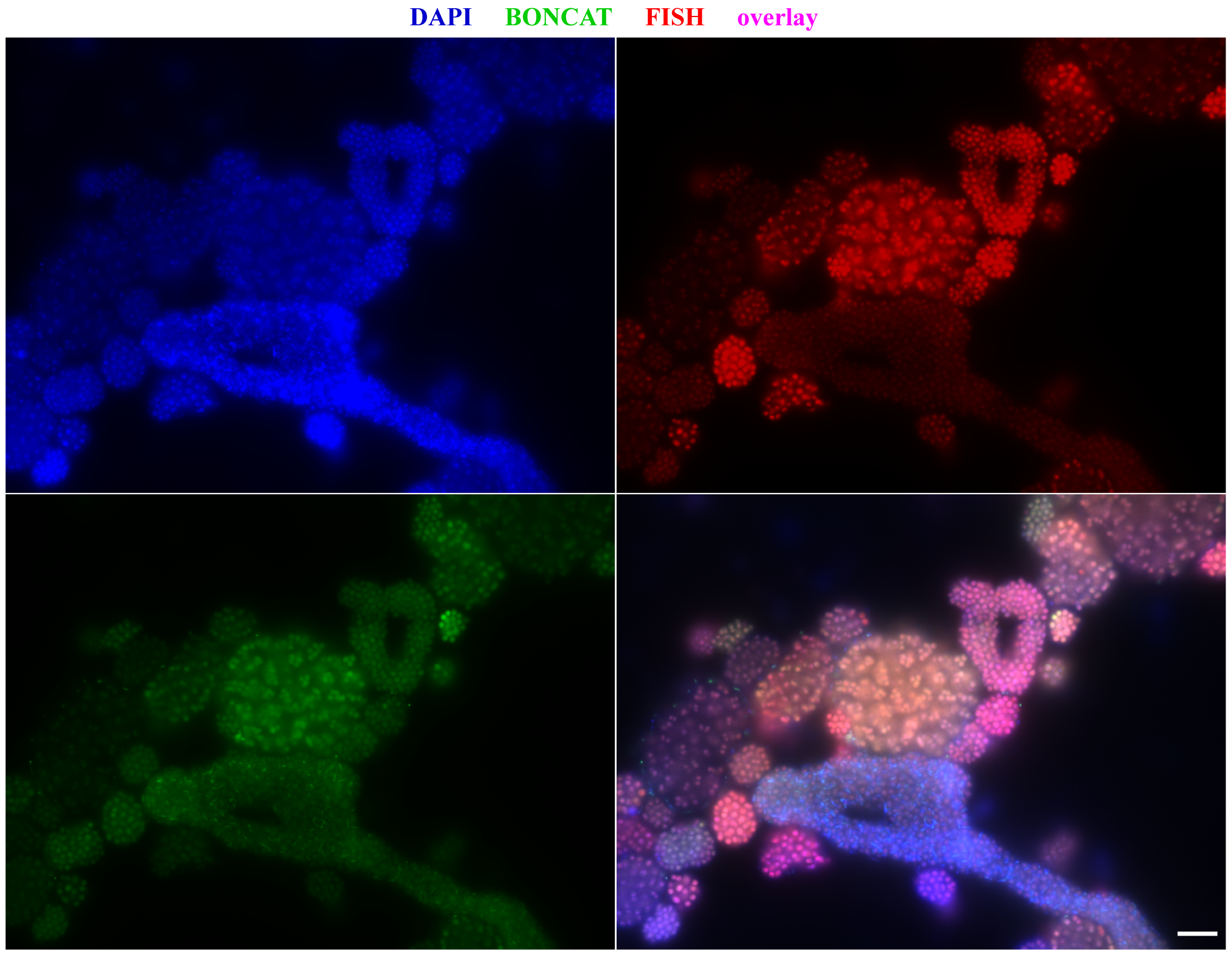

Supplement: Fig S8 — Visualization of clusters of protein-synthesizing gammaproteobacterial freshwater bacteria (identified via FISH-probe Gam42a (red), which was used with its competitor). The scale bar equals 20 μm. [file emi0016-2568-SD8.tif]
