# Supplementary material for: In situ visualization of newly synthesized proteins in environmental microbes using amino acid tagging and click chemistry
Source: Environ Microbiol. 2014 Apr 1;16(8):2568–90. doi: 10.1111/1462-2920.12436 (PMC4122687; doi:10.1111/1462-2920.12436)
Supplement: File S1 — liveBONCAT: Attempting to visualize protein synthesis in living cells. Strain-promoted click labeling of living microbes. [file emi0016-2568-SD10.doc]

**Supplementary Online Information**

***In situ* visualization of newly synthesized proteins in environmental microbes using amino acid tagging and click chemistry**

Roland Hatzenpichler^1,*^, Silvan Scheller^1^, Patricia L. Tavormina^1^, Brett M. Babin^2^, David A. Tirrell^2^, Victoria J. Orphan^1,*^

Divisions of ^1^Geological and Planetary Sciences and ^2^Chemistry and Chemical Engineering, California Institute of Technology, Pasadena, 1200 E. California Blvd, CA-91125, USA

**Results and Discussion**

liveBONCAT: attempting to visualize protein synthesis in living cells

Within the past years there has been growing interest in the recovery of intact cells from environmental samples, *e.g.* via pre-sorting microbial populations by fixation free rRNA-targeted FISH for genomic sequencing ([Yilmaz et al., 2010](#_ENREF_1)). If fluorescence labeling of AHA-containing proteins could be extended to living microorganisms it could offer a means of sorting anabolically active cells from complex samples and augment cultivation approaches by enabling pre-screening of samples for metabolisms of interest.

*E. coli* cultures that had been incubated in the presence or absence of AHA (1 mM) were analyzed via strain-promoted click chemistry. Cu(I)-catalyzed click chemistry was not tested due to the cytotoxic concentration of copper in the labeling buffer (100 μM). Results from liveBONCAT with viable *E. coli* cultures revealed only a small number (~1-2%) of fluorescently labeled cells (as compared to DAPI staining). Permeabilization of the cell membrane by Triton X-100 treatment increased the percentage of fluorescently labeled cells to ~10% of all DAPI stained cells. However, under both experimental conditions, *E. coli* cells exhibited atypical cell morphologies (Fig. S2), and the low concentration of cells relative to chemically fixed controls suggests cell lysis during labeling. When click chemistry mediated labeling was performed on aliquots of chemically fixed cells from the same culture, cells did not exhibit morphological abnormalities and >99% of DAPI-stained cells were fluorescently labeled (Fig. S2).

To test the viability of cells that had been subjected to liveBONCAT, we inoculated cell aliquots into growth media. After overnight incubation, cells from both experimental setups (without permeabilization and with Triton X-100 addition) had grown to the same optical density as control cells from the same AHA-treated culture which had not undergone the dye-labeling protocol (not shown). These preliminary results suggest that it may be possible to fluorescently label microbial cells *in vivo*, although the existing liveBONCAT protocol requires further testing and optimization to enhance the percentage of cell labeling.

**Experimental Procedures**

Strain-promoted click labeling of living microbes

After the incubation of *E. coli* cells in the presence or absence of 1 mM AHA, cultures were harvested via centrifugation and washed with 1x PBS (as described above), before being resuspended in 1x PBS. Equal volumes of this solution were then subjected to four different treatments, which were followed by incubation in 100 mM 2-chloroacetamide and a 30 min click labeling reaction using 1 μM of dye DBCO-PEG4-Carboxyrhodamine 110 at RT in the dark. These treatments were: (i) fixation and permeabilization for 5 min at 4 °C in 96% ethanol; (ii) fixation in 3% FA for 1 h at RT, followed by centrifugation and resuspension in 1x PBS (as described above); (iii) treatment with 0.05% Triton X-100 in 1x PBS for 5 min at RT, followed by a wash step and resuspension in 1x PBS. (iv) one cell aliquot was directly labeled without any pretreatment. After fluorescent labeling, cells were harvested via centrifugation (2 min, 16,100 g at RT), resuspended in 1x PBS and an aliquot microscopically analyzed.

The remaining volume of BONCAT-labeled cells was inoculated into 3 mL aliquots of M9 minimal medium supplemented with glucose (as described above) and incubated overnight (16 h) at 37 °C at 150 rpm horizontal shaking. In addition, non-inoculated controls, controls using chemically fixed BONCAT-labeled cells, as well as incubations of AHA-labeled cells that had not been subjected to fluorescent labeling were performed. The next day, optical densities were comparatively measured.

**References**

Yilmaz, S., Haroon, M.F., Rabkin, B.A., Tyson, G.W., and Hugenholtz, P. (2010) Fixation-free fluorescence *in situ* hybridization for targeted enrichment of microbial populations. *ISME J* 4: 1352-1356.
